# Evidence for alcohol-mediated hemolysis and erythrophagocytosis

**DOI:** 10.1016/j.redox.2025.103742

**Published:** 2025-06-26

**Authors:** Chaowen Zheng, Siyuan Li, Johannes Mueller, Cheng Chen, Huanran Lyu, Guandou Yuan, Ane Zamalloa, Lissette Adofina, Parthi Srinivasan, Krishna Menon, Nigel Heaton, Stephan Immenschuh, Ines Silva, Vanessa Rausch, Seddik Hammad, Steven Dooley, Shilpa Chokshi, Antonio Riva, Songqing He, Sebastian Mueller

**Affiliations:** a Division of Hepatobiliary Surgery, The First Affiliated Hospital of Guangxi Medical University, Nanning, Guangxi, China; b Center for Alcohol Research, University of Heidelberg, Heidelberg, Germany; c Laboratory of Liver Diseases, National Institute on Alcohol Abuse and Alcoholism, National Institutes of Health, Bethesda, MD, USA; d Institute King's College Hospital NHS Foundation Trust, Denmark Hill, London, SE5 9RS, United Kingdom; e Institute of Transfusion Medicine and Transplant Engineering, Hannover Medical School, Hannover, Germany; f Molecular Hepatology Section, Medical Faculty Mannheim, Heidelberg University, Germany; g Department of Forensic Medicine and Veterinary Toxicology, Faculty of Veterinary Medicine, South Valley University, Quena, Egypt; h Peninsula Medical School, Faculty of Health, University of Plymouth, Plymouth, United Kingdom; i The Roger Williams Institute of Liver Studies, School of Immunology and Microbial Sciences, Faculty of Life Sciences and Medicine, King's College London, Foundation for Liver Research, and King's College Hospital, London, United Kingdom

**Keywords:** Alcohol-related liver disease, Alcoholic hepatitis, Bilirubin, Iron overload, Heme, Hemolysis, Eryptosis, Erythrophagocytosis, CD163, Red blood cell, Liver cirrhosis, Hemoglobin, Ferroptosis

## Abstract

Alcohol-related liver disease (ALD) is the most common liver disease worldwide; however, its underlying molecular mechanisms remain poorly understood. Here, we identify ethanol-mediated hemolysis and erythrophagocytosis as major contributors to ALD pathogenesis using both in vitro and in vivo models, as well as surrogate markers such as heme oxygenase-1 (HO-1) and CD163, a scavenger receptor for hemoglobin-haptoglobin complexes.

A key initial observation was the direct optical evidence of serum hemolysis in heavy drinkers, which diminished after one week of alcohol withdrawal. In parallel, soluble CD163 (sCD163) levels declined during alcohol detoxification correlating with liver damage and fibrosis stages. Moreover, red blood cells (RBCs) from heavy drinkers exhibited increased fragility under hemolytic stress. In ethanol-fed mice, we also observed serum hemolysis. Erythrophagocytosis in liver tissue was visualized by co-localization of CD163 and hemoglobin autofluorescence. In vitro studies confirmed that ethanol – at concentrations transiently present in the upper gastrointestinal tract during alcohol ingestion – directly induces hemolysis and primes RBCs for erythrophagocytosis through eryptosis, marked by externalization of phosphatidylserine. Both heme, released during hemolysis, and bilirubin, its degradation product, further amplified erythrophagocytosis at clinically relevant concentrations, suggesting a self-perpetuating cycle. The antioxidant N-acetylcysteine efficiently blocked ethanol-induced RBC priming for erythrophagocytosis.

In conclusion, alcohol triggers a cascade of hemolysis, eryptosis, and erythrophagocytosis that may contribute to the pathogenesis of alcoholic hepatitis and end-stage ALD. sCD163 could serve as a noninvasive marker of hemolysis-associated macrophage activation. This mechanism opens new avenues for antioxidant-based therapies and may help to explain typical iron abnormalities, including ferroptosis, and hyperbilirubinemia in ALD.

## Highlights

• Ethanol triggers hemolysis and erythrophagocytosis in human and mouse models of alcohol-related liver disease.
• Hemolysis markers (HO-1, CD163) correlate with liver damage and decline during alcohol withdrawal.
• RBCs from heavy drinkers are fragile and prone to hemolysis and eryptosis upon stress exposure.
• Bilirubin and heme amplify erythrophagocytosis at clinical levels, indicating a self-perpetuating cycle.
• NAC prevents ethanol-induced RBC priming and erythrophagocytosis by blocking eryptosis.

## Graphical abstract

**Figure:**
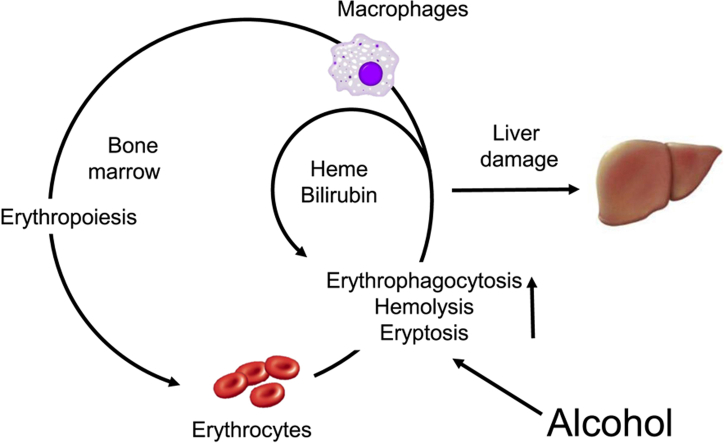

## Abbreviations

ALD: alcohol-related liver disease
BAC: blood alcohol concentration
BR: bilirubin
GGT: gamma-glutamyltransferase
Hb: hemoglobin
HMOX: heme oxygenase
HO-1: heme oxygenase isoform
Hp: haptoglobin
Hpx: hemopexin
Htc: hematocrit
LDH: lactate dehydrogenase
MCV: mean corpuscular volume of erythrocytes
NAC: N-acetylcysteine
NRF2: Nuclear factor erythroid 2-related factor 2
OD: optical density
PHZ: phenylhydrazine
PS: phosphatidylserine
RBC: red blood cell
sCD163: soluble CD163
SD: standard deviation

## 1 Introduction

Excessive alcohol consumption accounts for approximately half of all liver cirrhosis cases worldwide, establishing alcohol-related liver disease (ALD) as the most prevalent liver disease globally [1,2]. Despite its widespread occurrence and more than five decades of intensive research, the underlying mechanisms of ALD remain poorly understood, including its distinctive laboratory characteristics [2]. For instance, heavy alcohol consumption is often associated with elevated gamma-glutamyl transferase (GGT) levels, enlarged erythrocytes reflected by increased mean corpuscular volume (MCV), and elevated serum ferritin levels [3]. Additionally, liver biopsies indicate that approximately half of individuals with excessive alcohol intake exhibit hepatic iron accumulation, distributed almost evenly between macrophages and hepatocytes [[4], [5], [6]]. Evidence further highlights that especially binge drinking—characterized by consuming large quantities of alcohol within a short period of time—elevates the risk of liver disease independently of average alcohol intake [[7], [8], [9], [10]]. Binge drinking is also a significant contributor to acute liver injury and inflammation, often leading to alcoholic hepatitis [11]. So far, the molecular mechanisms underlying ALD and the harmful effects of binge drinking remain poorly understood.

Recent preliminary data from a 15-year prospective survival study in Caucasian heavy drinkers identified hemolytic anemia as a key long-term predictor of mortality [4,12]. The study primarily involved patients admitted for one-week alcohol detoxification without specific liver-related symptoms. Patients with high MCV had elevated ferritin, soluble CD163 (sCD163), and lactate dehydrogenase (LDH) levels, along with the highest mortality rate (∼30 %) during a mean follow-up of four years [4,12]. This high-MCV subgroup exhibited enhanced but ineffective erythropoiesis, without vitamin B12 or folic acid deficiencies, implicating alcohol's direct influence [13]. These findings suggest that hemolysis and erythrophagocytosis—the removal of damaged red blood cells (RBCs) by phagocytes—play a hitherto underestimated role in ALD [13]. Historically, hemolytic alterations like stomatocytosis in alcoholic patients were noted in case studies decades ago [[14], [15], [16]] and the Zieve syndrome, a rare form of alcohol-induced hemolysis, was first reported in 1958 [13,17]. Importantly, in this context, RBC can undergo not only hemolysis but also an apoptosis-like form of cell death known as eryptosis [18].

Hemolysis is considered highly toxic due to the release of hemoglobin that can release free heme, a pro-oxidant and pro-inflammatory compound [19]. To mitigate this toxicity, damaged or ruptured RBCs need to be efficiently cleared. A key pathway for this clearance is erythrophagocytosis, in which macrophages take up RBCs and neutralize the toxicity of hemoglobin. During this process, heme oxygenase (HO) encoded by the *HMOX* gene enzymatically converts heme into carbon monoxide (CO), biliverdin, and iron [[19], [20], [21], [22]]. Excess iron is stored in ferritin within hepatocytes and macrophages, with ∼90 % of the body's iron recycled from senescent erythrocytes [[23], [24], [25]]. Biliverdin is further reduced to bilirubin by biliverdin reductase [26]. Of the two HO isoforms, HO-1 is inducible and expressed at low levels in most tissues [22,27]. HO-1 is strongly upregulated in response to heme and is considered an adaptive mechanism to alleviate its toxicity [28,29]. The heme-dependent HO-1 regulation is mediated by an interplay of the nuclear repressor BTB-and-CNC homologue 1 (BACH1), a heme sensor protein with the master stress transcriptional regulator nuclear factor erythroid 2-related factor 2 (Nrf2) [30,31].

Although macrophages have been extensively studied in the context of ALD pathology [32], their role in recycling RBCs in ALD remains underexplored [33]. In addition to direct erythrophagocytosis, macrophages can also remove free hemoglobin and heme through distinct scavenging receptors. For instance, hemoglobin from lysed RBCs binds to haptoglobin (Hp), or after heme release, to hemopexin (Hpx). These complexes are internalized by macrophages through the hemoglobin-haptoglobin (Hb-Hp)-CD163 and heme-hemopexin (Heme-Hx)-CD91 pathways [34,35]. CD163, a scavenger receptor expressed on tissue macrophages [36], mediates clearance of Hb-Hp complexes, protecting against hemoglobin-induced oxidative damage during excessive hemolysis [37]. Levels of sCD163 reflect macrophage activity and have been linked to liver inflammation and fibrosis in clinical studies [[38], [39], [40]], though it remains unclear if this is directly tied to increased hemolysis.

Based on mortality data from ALD patients [12], we hypothesized that alcohol directly induces RBC hemolysis that, in turn, increases erythrophagocytosis and RBC turnover potentially contributing to liver damage. To test this hypothesis, we investigated markers of hemolysis and erythrophagocytosis in ALD patients and in an in vivo mouse model of chronic alcohol exposure. Moreover, an in vitro erythrophagocytosis model was established and applied to provide additional insights. Our findings highlight RBC recycling as a novel mechanism in ALD, contributing to hepatic iron overload and increased mortality.

## 2 Materials, methods and patients

### 2.1 Patients

Patients were prospectively enrolled as heavy drinkers (>80 g/day for males and >60 g/day for females) who primarily presented for approximately one week of in-hospital alcohol detoxification at Salem Medical Center in Heidelberg between 2007 and 2022. The mean alcohol consumption was 204 ± 148 g/day. All patients provided written informed consent prior to inclusion. The study protocols (435/2006, S385/2009, and S201/2015) were reviewed and approved by the Ethics Committee of the University of Heidelberg and adhered to the ethical guidelines of the 1975 Declaration of Helsinki. Upon admission, all patients underwent standard laboratory testing, abdominal sonography, and transient elastography. A subgroup of patients was scheduled for liver biopsy and hepatic CD163 mRNA expression analysis. Patient characteristics are detailed in Supplementary Table S1 and in Appendix of [2]. All participants were over 18 years old, and other causes of liver disease were excluded through serological screening for AMA, ANA, HIV, HBV, and HCV.

### 2.2 Ultrasound, transient elastography (TE) and CAP

Liver size, signs of cirrhosis, spleen size, ascites formation, and semi-quantitative liver steatosis (graded 0–3) were assessed using abdominal ultrasound. Liver stiffness was measured in kilopascals (kPa) using the FibroScan 502 platform (Echosens SA, Paris, France) with both the M and XL probes [41]. Hepatic fat content was evaluated with the FibroScan device by measuring the Controlled Attenuation Parameter (CAP), with values expressed in decibels per meter (dB/m), ranging from 100 to 400 dB/m [42].

Transient elastography (TE) was performed by physicians with at least 12 months of experience in abdominal ultrasound and TE, using the right lobe of the liver in an intercostal position according to established protocols [41]. Fibrosis stages were determined based on AST-adapted cutoff values as previously described [43].

### 2.3 Mouse model of chronic alcohol exposure

To study the effects of chronic alcohol exposure, we utilized an established mouse model incorporating a modified pair-fed Lieber-DeCarli diet [44] for three weeks, followed by a binge phase [45]. Briefly, 8-week-old male C57BL/6J mice were obtained from Charles River Laboratories and housed individually in ventilated cages under a 12-h light/dark cycle in specific pathogen-free (SPF) conditions. Female mice were not included due to the monthly blood loss that would critically interfere with read-out parameters of our study. The animal experiment was approved by the Animal Ethics Committee of Baden-Wuerttemberg (G155/15). In the control group, six wild-type C57BL/6J mice were fed an ad libitum Lieber-DeCarli control diet (Lieber-DeCarli'82, Bio-Serv, Product no. F1259SP) for three weeks, with access to a limited standard solid diet and water to acclimatize to tube feeding. In the chronic ethanol group, six mice received a Lieber-DeCarli ethanol liquid diet (5 % v/v, Lieber-DeCarli'82, Bio-Serv, Product no. F16797SP) for the same duration. After three weeks, the control animals received two isocaloric maltose dextrin gavages (45 % wt/vol) at 9-h intervals, while the chronic alcohol group received two acute ethanol gavages (31.5 % wt/vol) under the same schedule. The animals were sacrificed 9–12 h after the second gavage. Animal characteristics are detailed in Supplementary Table 2. Laboratory values were measured using routine techniques in the Central Laboratory (Zentrallabor) of the University Hospital Heidelberg. Visible optical signs of hemolysis next to jaundice or lipemia was also routinely scored in the Central Laboratory and scored.

### 2.4 Animal model of hemolysis

Six 8-weeks male mice with C57BL/6J background, obtained from Charles River Company, received two injections of the hemolytic agent phenylhydrazine (PHZ) (60 mg/kg body weight), one after 24 h and the last one after 48 h. A control group (n = 6) received saline injections. All animals were hosted in single ventilated cages in a 12-h day/night cycle under specific pathogen-free (SPF) conditions. The experiment was approved by the animal ethics committee in Baden-Wuerttemberg (G155/15).

### 2.5 Hemolytic in vitro model for human RBCs

Stress sensitivity to the PHZ was tested as previously described [46]. PHZ (10 mM) was incubated with 1:18 diluted blood for 60 min. The degree of hemolysis was determined by measuring hemoglobin concentration photometrically using a Helios Alpha Spectrophotometer (Unicam) in the supernatant of the red cell suspensions, following conversion to cyanmethemoglobin at 546 nm. For this assay, 0.5 mL hemolysate was incubated with 1.5 mL Drabkin's solution (5 mg potassium cyanide, 20 mg potassium hexacyanoferrate, 0.1 g sodium bicarbonate in 100 mL triple-distilled water) for 20 min. Blood samples from six healthy volunteers and six ALD patients undergoing alcohol detoxification at Salem Medical Center Heidelberg between 2017 and 2018 were used for the analysis.

### 2.6 Visible signs of hemolysis

**Fig. 1:**
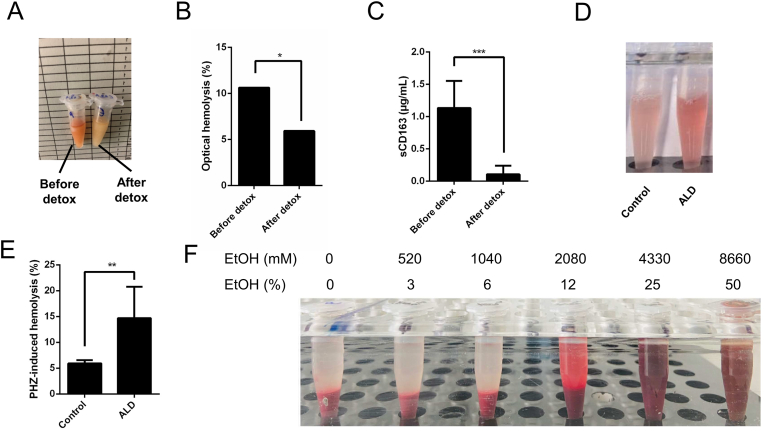
Signs of hemolysis, enhanced heme turnover and increased RBC fragility in heavy drinkers. **A)** Example of macroscopic optical signs of hemolysis in a serum sample before and one week after alcohol detoxification. **B)** Significantly decreased signs of optical hemolysis determined by optical inspection in patient sera from heavy drinkers after alcohol withdrawal (see methods section). Frozen serum samples from n = 439 heavy drinkers prior and one week after alcohol detoxification were used. Of note, the data suggest that one week of alcohol detoxification already improves ethanol-mediated RBC fragility. **C)** Serum sCD163 levels (soluble hemoglobin-haptoglobin scavenging receptor) measured by ELISA before and one week after alcohol detoxification in n = 72 heavy drinkers. Shown is median and interquartile range. **D and E)** RBCs of heavy drinkers are more fragile in response to hemolytic stress by the hemolytic agent phenylhydrazine (PHZ) or mechanically during blood taking. RBCs from both heavy drinkers and healthy volunteers (each cohort n = 6) were treated in vitro with PHZ for 60 min and hemolysis was measured by absorption spectroscopy in the supernatant (see methods section). **F)** Direct exposure of human RBCs with ethanol (EtOH) for 24 h. Note, that hemolysis or modification only occurs at quite high levels of ethanol (ca. 10 %) which, however, may be achieved during binge drinking in some compartments using high percentage liquors or wine. Exposure time can be as short as 60min to lyse RBCs. ∗P < 0.05, ∗∗P < 0.01, ∗∗∗P < 0.001, ∗∗∗∗P < 0.0001.

Signs of visible hemolysis in 439 frozen patient sera were determined by the presence or absence of red color in the serum after centrifugation as is routine practice in medical laboratories (see Fig. 1B). Hemolysis was detected by two independent examiners that were blinded to the study.

### 2.7 Cell culture and isolation of human blood monocytes

The American Type Culture Collection (ATCC, Manassas, VA, USA) provided the immortalized human monocyte THP-1 cells. These cells were cultured in RPMI-1640 medium supplemented with 10 % fetal bovine serum and 25 mM glucose (Gibco, Thermo Fisher Scientific, Waltham, MA, USA). To induce differentiation into monocyte-derived macrophages (MDM), THP-1 monocytes were seeded in 12-well plates and treated with 100 ng/ml of phorbol myristate acetate (PMA) for 24 h. After differentiation, the cells were washed and incubated in fresh media for 24 h prior to experimentation.

Human primary monocytes were isolated and purified from peripheral blood mononuclear cells (PBMCs) of buffy coats obtained from healthy blood donors using a Biocoll gradient (Biochrom, Berlin, Germany). After isolation, PBMCs were resuspended in RPMI medium and seeded at a density of 2x10^6^ cells/well in non-coated 12-well plates. The monocytes were obtained by selective adherence after 1 h of incubation and cultured over 7 days in RPMI medium supplemented with 10 % FBS to allow the differentiation into monocyte-derived macrophages.

### 2.8 Co-culture, treatments and in vitro erythrophagocytosis model

A direct co-culture system of THP-1-derived macrophages and RBCs was established by seeding THP-1 monocytes in 12-well plates at a density of 0.3 × 10^5^ cells/well and inducing differentiation with PMA. After 24 h, RBCs were added to the wells. RBCs were either oxidized using CuSO_4_ (0.2 mM) and ascorbate (5 mM) for 2 h, followed by washing with 0.9 % sodium chloride solution, or primed for erythrophagocytosis using different concentrations of ethanol (100 mM–1600 mM) [47,48]. In several experiments, the cells were exposed to varying concentrations of alcohol for 2–24 h. Experimental validation confirmed that alcohol concentrations remained stable, with less than a 10 % decrease due to evaporation (not shown).

### 2.9 Phagocytosis assay

Erythrophagocytosis was assessed using a commercial kit (CytoSelect™ 96-Well Phagocytosis Assay (Red Blood Cell) Kit, MyBiosource, MBS168645), which employs the phagocytosis inhibitor cytochalasin D. THP-1 monocytes were seeded into 96-well plates at a density of 2 × 10^4^ cells/ml and differentiated into macrophages. Subsequently, the cells were treated with varying concentrations of cytochalasin D and co-cultured with either 1 % oxidized RBCs (oxiRBCs) or RBCs pretreated with 800 mM ethanol (EtOH) for 24 h. The assay was performed according to the manufacturer's protocol, and absorbance was measured at 630 nm using a microplate reader.

### 2.10 Heme measurement

Heme concentrations were measured before and after a 24-h co-culture of THP-1-derived macrophages with 0.5 % oxiRBCs. Supernatants were collected at both time points, and heme levels were quantified using a colorimetric heme assay kit (Abcam, ab272534) according to the manufacturer's instructions.

### 2.11 Live-cell imaging system

Real-time videos of the uptake of ethanol-primed RBCs by THP-1 macrophages were captured using the IncuCyte® S3 Live-Cell Analysis System (Essen BioScience, Royston, UK; Cat. No.: 4647). This platform enables continuous acquisition of full-well images and videos directly from live-cell cultures. Images were recorded at 2-min intervals, which represents the highest available time resolution of the system.

### 2.12 Precision cut living liver slices

Human precision-cut liver slices (PCLS) were collected from peritumoral tissue of colorectal liver metastases, along with blood samples from the same patients. Sample collection and preparation were conducted as described previously, following informed consent according to the Declaration of Helsinki and local ethical approval (REC reference 17/NE/0340; IRAS project ID 222302; October 31, 2017) [49]. Briefly, the collected human liver tissue was preserved in sterile University of Wisconsin solution (ViaSpan; Bridge to Life Ltd., London, UK) until slicing. PCLS (approximately 5 mg in weight and 250 μm thick) were prepared using a Krumdieck tissue slicer (Alabama R&D, Munford, AL, USA). Following slicing, the PCLS underwent a 2-h recovery step in culture before being transferred to fresh media until the next morning, which was designated as the start of the experiment (Day 0). The total ex vivo culture duration was four days (Day 0 to Day 3). Slices were cultured in a 12-well plate (Nunc, Thermo Fisher Scientific, Oxford, UK) with 1.5 mL of William's E Medium (Thermo Fisher Scientific, Oxford, UK) supplemented with 5 % Human AB serum (Pan-Biotech, Wimborne, UK), penicillin/streptomycin (Thermo Fisher Scientific, Oxford, UK), 2 mM glutamine (Thermo Fisher Scientific, Oxford, UK), ITS (10 mg/L insulin, 5.5 mg/L transferrin, and 6.7 μg/L sodium selenite; Thermo Fisher Scientific, Oxford, UK), 1 nM epidermal growth factor (Thermo Fisher Scientific, Oxford, UK), 100 nM glucagon (Merck, Gillingham, UK), and 1 μM corticosterone (Merck, Gillingham, UK).

### 2.13 Chemicals, reagents, and H_2_O_2_ exposure protocol

PMA (100 ng/ml), CuSO_4_ (0.2 mM), ascorbate (5 mM), hemin, and bilirubin were purchased from Sigma-Aldrich (Taufkirchen, Germany). H_2_O_2_ exposure was conducted using the glucose oxidase (GOX) as previously described [51,52]. Intracellular macrophage catalase served to maintain peroxide steady-state levels. For the protocol, THP-1 cells were seeded in 12-well plates with 10 % FCS medium and incubated for 24 h, followed by 24 h GOX treatment with different concentrations.

### 2.14 Immunofluorescence and confocal microscopy

Immunofluorescence staining of frozen mouse liver sections was performed to study the expression of CD163. Frozen liver sections were incubated overnight at 4 °C with an Anti-CD163 antibody (PR19518) diluted 1:200. Following three washes with PBS, the slides were incubated in the dark for 1 h with Alexa Fluor 647-conjugated donkey anti-goat secondary antibody (#711605152) diluted 1:1000. After a final rinse with PBS, the slides were counterstained with DAPI (Sigma, St. Louis, MO, USA) and analyzed using a fluorescence microscope (ZEISS Axio Scan. Z1, Carl Zeiss Microscopy GmbH, Germany).

Hemoglobin autofluorescence was detected using the Alexa Fluor 488 channel. Preliminary data indicated that hemoglobin autofluorescence displayed a staining pattern consistent with that of anti-hemoglobin antibodies. Utilizing autofluorescence reduced nonspecific signals caused by cross-reactivity and the use of excessive antibodies, enhancing staining specificity. Final settings and conditions were as follows: amplification 20X, CD163 antibody: EPR19518, Abcam, 1:500 dilution, Secondary antibody: Alexa Fluor® 647-Donkey Anti-Rabbit IgG (H + L) (min X), jacksonimmuno.com, 711-605-152, RBC autofluorescence: Red, channel 488. We also studied the expression of phosphatidylserine (PS) in RBCs. In brief, RBCs were incubated with Annexin V (1:200 dilution) polyclonal antibody overnight at 4 °C. After washing three times with PBS, the slides were incubated with Alexa Fluor 647-conjugated donkey anti-rabbit IgG in 1:500 dilution.

### 2.15 RNA isolation, cDNA synthesis and real time quantitative PCR analysis

Total RNA from cells and animal tissues was isolated by Trizol Reagent (Thermo Fisher, 15,596,018) according to the supplier's standard protocol. Reverse transcription was performed as reported previously [50]. PCR was performed either by dual labeled probes or the SYBR green method. For the SYBR green method, primers (see Suppl. Table 3A) were synthesized at Eurofins MWG Operon (Ebersbach, Germany), For UDP probes, probes and primers were designed by and bought from the Universal Probe Library (LifeScience, Roche Molecular Systems). Real-time quantitative PCR (qRT-PCR) was performed and read by LightCycler PCR system (Roche Molecular Systems, Inc.). Results were analyzed using the double delta Ct method [51].

### 2.16 Western blotting

Cells or tissue were harvested in RIPA buffer plus 1 × Complete protease inhibitor with EDTA (Roche Applied Sciences) on ice. Western Blotting was performed as described previously [50]. Primary and secondary antibodies are listed in Suppl. Table 3B. Immune-reactive bands were detected by Odyssey CLx imaging system. Band intensities were quantified using ImageJ (NIA, USA) for further statistical analysis.

### 2.17 Enzyme-linked immunosorbent assay (ELISA)

High-binding surface microplates for ELISA were purchased from Corning Company (Product No. 9018). A direct ELISA assay was established using a human CD163 Elisa kit (Biorbyt Company, orb 390857). ELISA was performed following the manufacturer's instructions. Patient sera were diluted tenfold prior to use. Each sample was measured three times on the same plate, and the average value was taken. In 72 patients, sCD163 serum levels could also be measured after one week of alcohol detoxification. Antibodies are listed in Suppl. Table 3B.

### 2.18 MTT assay

The MTT assay was conducted to evaluate cell viability [52,53]. THP-1 monocytes, differentiated using PMA, were seeded into 96-well plates at a density of 4 × 10^4^ cells per well and incubated overnight. Following treatment with varying concentrations of ethanol (EtOH) or a 24-h recovery period, 20 μL of MTT solution (5 mg/mL in PBS) was added to each well. The plates were then incubated at 37 °C for 4 h. After incubation, the resulting formazan crystals were dissolved in 150 μL of dimethyl sulfoxide (DMSO), and the absorbance was measured at 570 nm using a microplate reader (FLOUstar Optima, BMG Labtech, Germany). Cell viability was expressed as a percentage relative to the control group.

### 2.19 Statistical analysis

All data are presented as mean ± standard deviation (SD). Statistical analyses were conducted using GraphPad Prism 6 (GraphPad Software Inc., USA), Excel (Microsoft, USA), or Python 3 (Python Software Foundation, https://www.python.org/). For descriptive statistics, means and standard deviations or medians and interquartile ranges were calculated as appropriate.

Correlation analysis was performed using the Spearman rank-order coefficient. Statistical significance was evaluated using the unpaired *t*-test, Mann-Whitney *U* test, or Fisher's exact test, as appropriate. For comparisons involving multiple groups, one-way ANOVA followed by Tukey's or Dunnett's post hoc test for multiple comparisons was applied. Significance levels were denoted as follows: n. s. (not significant), ∗ (p < 0.05), ∗∗ (p < 0.01), ∗∗∗ (p < 0.001).

## 3 Results

### 3.1 Evidence for increased hemolysis and erythrophagocytosis in heavy drinkers

We conducted a retrospective, blinded analysis of serum samples from heavy drinkers collected before and after alcohol detoxification to identify optical signs of hemolysis (Fig. 1A and B). A total of 439 paired samples, representing pre- and post-detoxification states after one week of alcohol withdrawal, were obtained from the Heidelberg cohort (refer to Patients and Methods). Visible optical signs of hemolysis were observed in 10 % of samples prior to detoxification, decreasing significantly to 5 % (P < 0.05) following detoxification (Fig. 1B).

Serum levels of sCD163, a scavenger receptor for the hemoglobin-haptoglobin complex, were measured in 72 heavy drinkers using ELISA (Fig. 1C). Initial levels exceeded 1000 ng/mL but significantly decreased to 208 ng/mL after detoxification. Prototypical hemolysis markers, including serum LDH and ferritin, also showed significant reductions post-detoxification (Suppl. Fig. 1).

To further investigate RBC fragility under hemolytic stress and ethanol exposure, in vitro experiments were conducted using RBCs from heavy drinkers and healthy volunteers (n = 6 per group). RBCs were treated with PHZ for 60 min, and hemolysis was quantified via measuring Hb in the supernatant. In Fig. 1D and E it is demonstrated that RBCs from heavy drinkers exhibited increased fragility when exposed to PHZ-induced hemolytic stress compared to those from healthy individuals. Additionally, direct exposure of human RBCs from healthy controls to ethanol under iso-osmolar conditions revealed that high ethanol concentrations (>12 %/2000 mM) are required to cause rapid hemolysis (Fig. 1F).

In conclusion, we demonstrate that heavy drinkers exhibit both optical and biochemical signs of hemolysis, including elevated sCD163, which decreased rapidly after alcohol detoxification. RBCs from heavy drinkers are more sensitive to hemolytic stress, and while ethanol can directly induce hemolysis, this occurs only at high concentrations.

### 3.2 Serum hemolysis markers highly correlate with liver damage, iron and mortality in heavy drinkers

Supplementary Table 4A presents the Spearman rho correlations of LDH and hemoglobin with various parameters, confirming their association with hemolysis. LDH shows strong positive correlations with ferritin, sCD163, and indirect bilirubin, while demonstrating negative correlations with hemoglobin, haptoglobin, and RBC count. Additionally, LDH is positively correlated with liver damage markers such as AST, ALT, and M30. Clinically, LDH is associated with liver stiffness and mortality, whereas hemoglobin exhibits negative correlations with these parameters. The inverse relationship between LDH and hemoglobin (r = −0.137, p = 3.33E-04) underscores the hemolytic nature of low hemoglobin levels. Notably, both markers showed strong associations with long-term mortality (P < 0.0001 in Mann-Whitney *U* test), emphasizing the critical role of hemolytic anemia in ALD.

Correlation of sCD163 is shown in Supplementary Table 4B. For better readability, positive and negative correlations are presented in separate columns. sCD163 is strongly positively correlated with hemolysis markers, including LDH and indirect bilirubin, as well as liver damage markers such as AST and M30 [54]. It also correlates positively with liver fibrosis parameters (e.g., liver stiffness [41]) and bile acid levels. Notably, sCD163 shows negative correlations with hemolysis markers like hemopexin and haptoglobin (carriers of free heme or hemoglobin), the iron carrier transferrin, and liver synthesis markers such as albumin, which is also an important carrier of bilirubin. Furthermore, sCD163 negatively correlates with hemoglobin and RBC count. In conclusion, these findings underscore the strong association between anemia, hemolysis, erythrophagocytic activity, and liver damage.

### 3.3 Hepatic expression of CD163 mRNA in heavy drinkers and human liver slices

Hepatic *CD1*63 mRNA expression was analyzed in 30 liver specimens from ALD patients (Supplementary Table 5). *CD1*63 mRNA levels significantly correlated with Toll-like receptor 4, oxidized peroxiredoxin 2, *Nrf2*, ferroptosis marker GPX4 and hepatocyte iron. While a positive correlation with *HO-1/HMOX1* was observed, it was not statistically significant. *CD1*63 mRNA was also positively associated with fibrosis severity (histological stage and elastography-measured liver stiffness) and bilirubin levels. Interestingly, a negative correlation was found with AST and ALT, potentially reflecting decreased liver damage in advanced fibrosis stages.

**Fig. 2:**
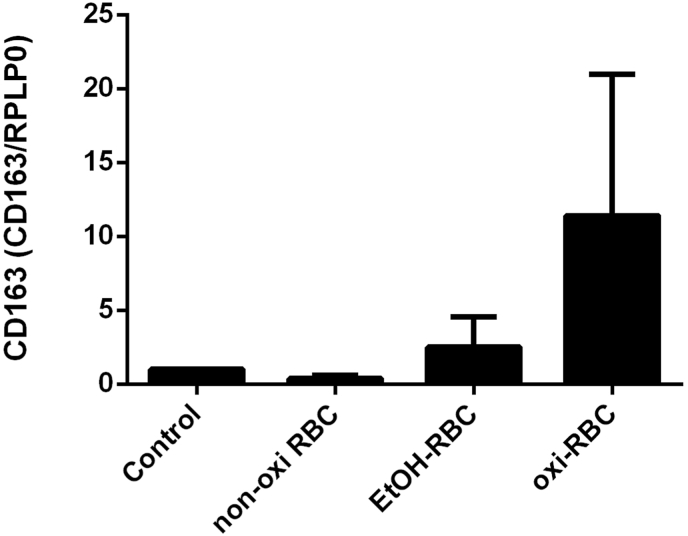
CD163 mRNA expression in living precision cut liver slices (PCLS) treated with different RBC conditions. This figure shows CD163 mRNA expression in PCLS treated with normal non-oxidized RBCs, alcohol-treated RBCs (200 mM), and CuSO_4_-oxidized RBCs. Results are presented as mean with min-max range (*n* = 3 per group). Liver slices were obtained from healthy liver tissue following liver resections for colorectal liver metastases.

To explore *CD1*63 mRNA regulation, PCLS were utilized. These PCLS derived from non-cancerous peritumoral tissue of colorectal liver metastases, were cultured ex vivo for four days (Day 0–Day 3). The viability of PCLS using this protocol was previously validated in detail (LDH release, ATP content, histology) and remained stable over 4 days, as published earlier by our group [49]. As shown in Fig. 2, *CD1*63 mRNA expression increased significantly after 24-h exposure to ethanol-pretreated or oxidized RBCs. Ethanol pretreatment tripled *CD1*63 mRNA levels, while Cu^2+^-mediated oxidation caused a nearly tenfold increase. In conclusion, in humans, CD163 mRNA is correlated with oxidative stress, fibrosis, liver injury, and hepatocellular iron accumulation. It is further inducible in living human precision-cut liver slices in response to alcohol.

### 3.4 Enhanced erythrophagocytosis in a chronic ethanol feeding model

**Fig. 3:**
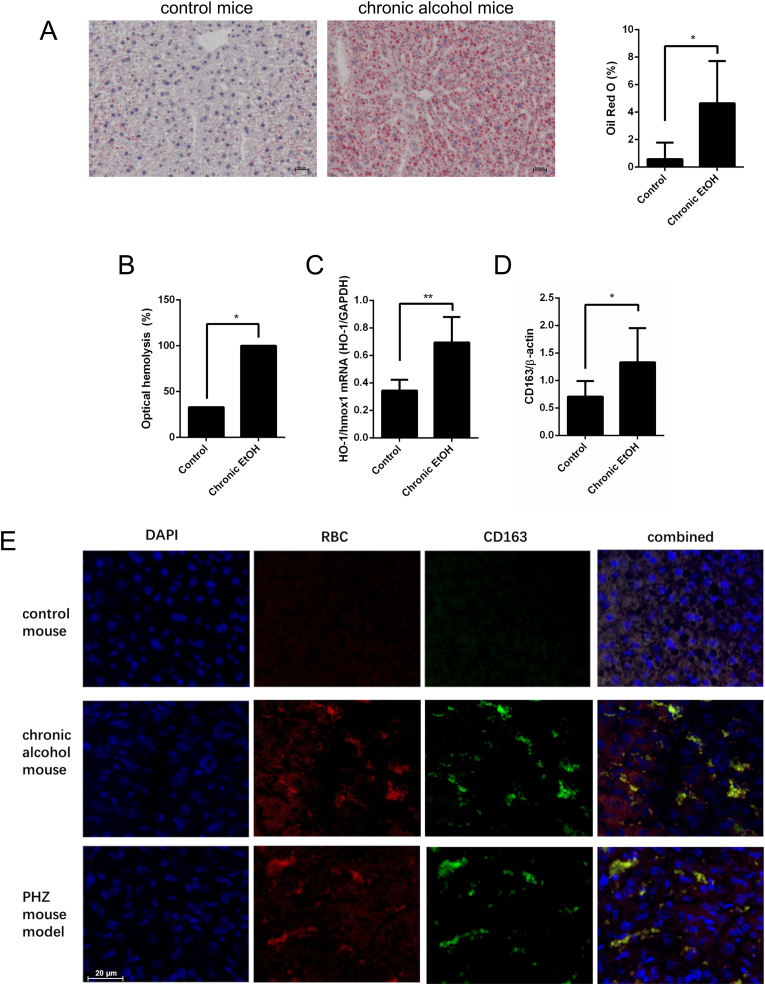
Signs of hemolysis and induction of the hepatic hemoglobin-haptoglobin scavenger receptor CD163 in a chronic alcohol exposure model. **A**) Three weeks of 5 % v/v ethanol diet with 2 acute alcohol gavages [45] cause significant hepatic steatosis and characteristic patterns of serum markers (see Suppl. Table 3). Representative example of oil red O stain (steatosis) and HE background stain. Note that no histological inflammation and fibrosis is seen in this setting. n = 6 per group. ∗P < 0.05, mean and SD. **B)** Presence of optical signs of hemolysis determined by optical inspection in serum samples from ethanol-treated mice (each group n = 6). ∗P < 0.05 **C)** Significant induction of *HO-1/HMOX1* mRNA and **D)** CD163 protein expression in mouse liver after three weeks of chronic alcohol feeding (each group, n = 6 mice, 2 measurements per mouse). ∗P < 0.05, ∗∗P < 0.01, mean and SD. **E)** Confocal images of CD163 and RBC autofluorescence staining in control and a chronic alcohol mouse model of three weeks of chronic alcohol exposure. For comparison, the images of a hemolysis mouse model using hemolytic agent phenylhydrazine injections over 48 h are also shown.

To investigate the relationship between erythrophagocytosis and alcohol, we utilized a chronic ethanol feeding model [45]. Male B6 mice were exposed to ethanol for three weeks, with two alcohol gavages at the end of the period, mimicking binge drinking that caused histopathological signs of fatty liver (Fig. 3A) and elevated transaminase levels (Supplementary Table 2). Serum analysis revealed a higher prevalence of hemolysis in ethanol-fed animals (Fig. 3B). Upregulation of *Hmox-*1 mRNA (Fig. 3C) indicated enhanced hepatic heme degradation, consistent with previously reported elevated HO-1 protein levels [55].

*CD1*63 mRNA expression was significantly increased in the ethanol cohort (Fig. 3D), with Western blotting confirming elevated hepatic CD163 protein levels (Supplementary Fig. 2). Corresponding HO-1 protein expression has been reported earlier [55]. Immunofluorescence analysis, using DAPI as a nuclear counterstain and hemoglobin autofluorescence, visualized macrophage-specific CD163 expression (Fig. 3E). Hemoglobin autofluorescence closely matched the pattern of anti-hemoglobin antibodies, confirming minimal cross-reactivity. In vivo erythrophagocytosis was verified by co-localization of hemoglobin autofluorescence with CD163 expression in ethanol-exposed animals. A hemolysis model using PHZ served as a positive control.

In summary, this chronic ethanol exposure model demonstrates hemolysis and increased hepatic CD163 expression. The co-localization of CD163 with hemoglobin autofluorescence confirms enhanced erythrophagocytosis associated with alcohol exposure.

### 3.5 sCD163 levels are increased in patients with liver fibrosis

**Fig. 4:**
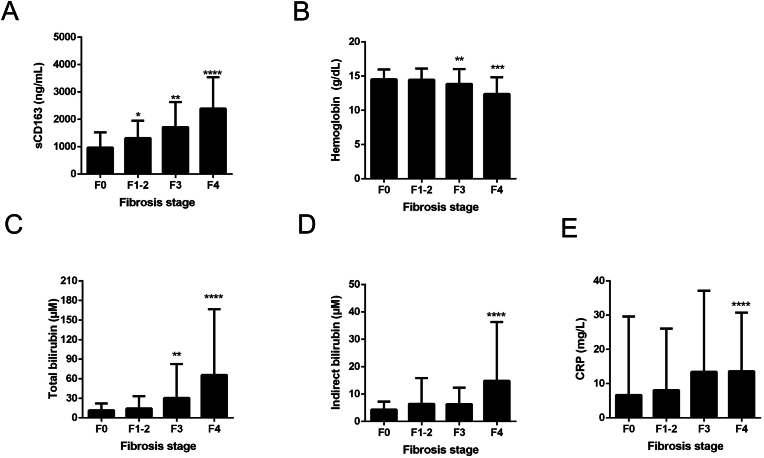
Enhanced heme turnover increases at higher fibrosis stages. Shown are **A)** serum levels of sCD163 **B)** hemoglobin **C)** total bilirubin, **D)** indirect bilirubin and **E)** CRP. Shown is mean and 95 % C·I. Asterix represent P values vs the F0 group. Values were adjusted for multiple comparisons using Dunnett's method. ∗P < 0.05, ∗∗P < 0.01, ∗∗∗P < 0,001, ∗∗∗∗P < 0.0001. Note that higher fibrosis stages not only cause enhanced erythrophagocytosis (sCD163) and anemia but also elevated indirect bilirubin as a direct sign of hemolysis. Data are from n = 228 heavy drinkers.

We next studied signs of erythrophagocytosis in n = 228 heavy drinkers using serum sCD163 as a function of fibrosis stage. As shown in Fig. 4A, sCD163 serum levels continuously increased with increased levels of fibrosis. Fig. 4B, C and D show the corresponding levels of hemoglobin, total and indirect bilirubin the latter being a direct surrogate marker of hemolysis. These data directly indicate signs of hemolysis (elevated indirect bilirubin in advanced fibrosis stages) and continuously decreasing hemoglobin values. Of note, sCD163 rapidly normalized in all fibrosis stages during a mean alcohol withdrawal time of 7 days (Suppl. Fig. 3). Post-detox levels of sCD163 appeared to be slightly lower in patients with advanced fibrosis compared to patients with earlier stages, although this difference was not significant (Suppl. Fig. 3). Taken together, these data strongly suggest that ethanol-mediated hemolysis/erythrophagocytosis is more pronounced in patients with advanced liver fibrosis.

### 3.6 Establishment of an in vitro model to study human erythrophagocytosis

**Fig. 5:**
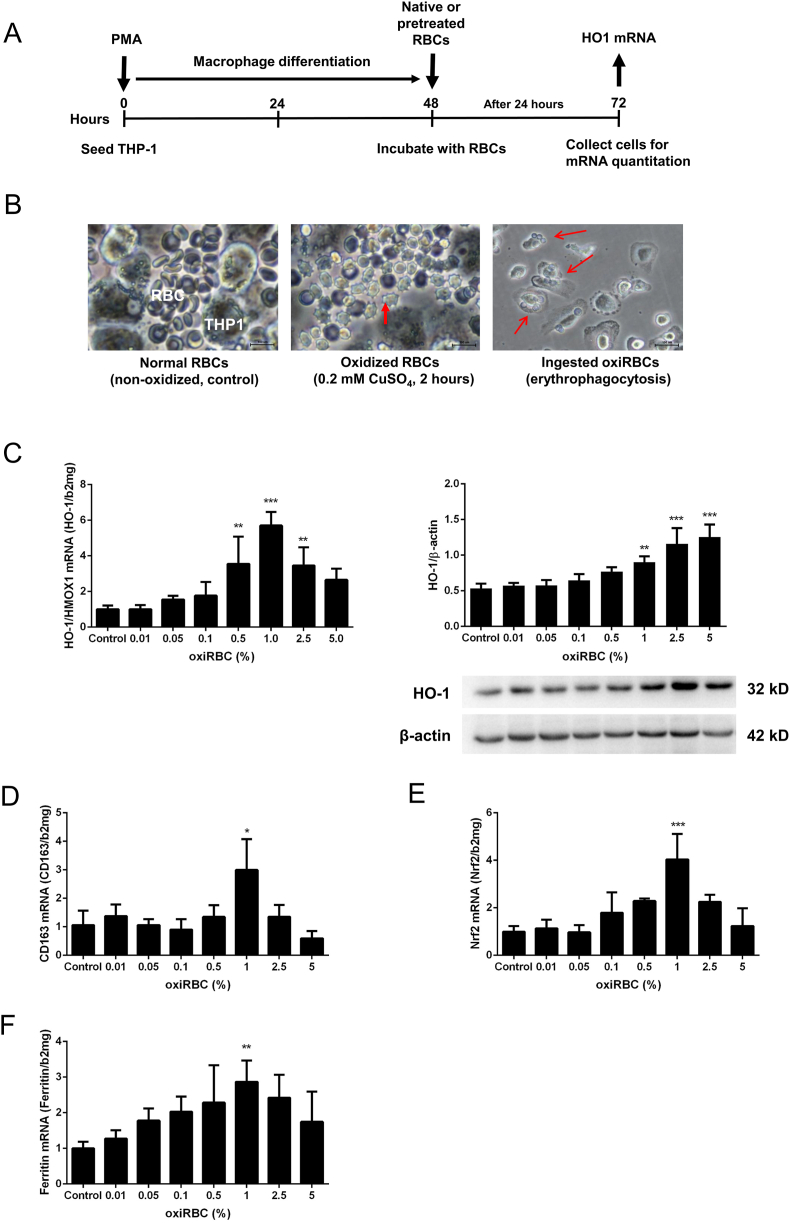
Erythrophagocytosis in vitro of oxidized human erythrocytes. A) Experimental design to investigate erythrophagocytosis in vitro using human RBC and differentiated THP-1 macrophages. THP-1 monocytes were differentiated to THP-1 macrophages through PMA treatment over 24 h. THP-1 macrophages were then cocultured with isolated human RBC pretreated with ascorbate/copper sulfate for 120 min. **B)** Left panel: Control RBCs and co-cultured human THP-1 macrophages. Middle panel: Morphological changes (spur cells (acanthocytes), red arrow) of RBCs in the presence of copper sulfate-induced oxidative stress after 120 min. Right panel: Erythrophagocytosis of oxidized human erythrocytes (red arrow, oxidized by copper sulfate) by THP-1 macrophages. Representative images of n = 10. **C)** Oxidized RBC induce erythrophagocytosis as measured by *HO-1/HMOX1* mRNA (left panel) and protein expression (right panel) in a dose dependent manner. RBC were oxidized by CuSO_4_ for 2 h and then co-culture different doses of oxidized RBC with macrophages for 24 h. **D)** Expression of *CD1*63 mRNA and **E)** Expression of *Nrf2* mRNA, an important transcription factor upstream of HO-1. **F)** Expression of Ferritin heavy chain mRNA. mRNA was quantified by quantitative real-time PCR using triplicates and the results are represented as mean of mRNA levels normalized to β2-macroglobulin ±SD. ∗P < 0.05, ∗∗P < 0.01 (vs control).

Based on our preliminary studies [4], an in vitro model was established to investigate ethanol-mediated mechanisms of erythrophagocytosis. As illustrated in Fig. 5A, immortalized human THP-1 monocytes, which grow in suspension culture, were differentiated into adherent macrophages using PMA for 48 h [56,57]. These macrophages were then exposed to either untreated human RBCs (Fig. 5B, left panel, controls) or oxidized RBCs pretreated with ascorbate and CuSO_4_ (Fig. 5B, middle panel). As shown in Fig. 5B (right panel), only oxidized RBCs were rapidly ingested by erythrophagocytosis, leading to RBC accumulation within THP-1-derived macrophages. In addition, oxidative stress induced by CuSO_4_ triggered spur-like (acanthocytic) transformations in RBCs (Fig. 5B, middle panel).

Supplementary Fig. 5A provides further images of oxidized RBC uptake, showing multiple vacuoles and enlarged macrophages. In contrast to hepatocyte-mediated erythrophagocytosis [33], RBCs in macrophages were continuously digested rather than accumulating. Phagocytosis was confirmed using a cytochalasin D-based assay, revealing a dose-dependent inhibition (see Supplementary Fig. 5B). The elimination of RBCs from the surrounding medium was also evident (see Supplementary Fig. 5C). Supplementary Fig. 5D displays a sequence of images from live-cell video microscopy, capturing ethanol-primed RBC uptake by THP-1-derived macrophages at 2-min intervals; however, direct visualization of engulfment events remained infrequent due to temporal resolution limits. Erythrophagocytosis could be also confirmed in human primary monocytes (Supplementary Fig. 5E).

Erythrophagocytosis of oxidized RBCs led to strong induction of HO-1/HMOX1 mRNA, detectable at a hematocrit of 0.1 % and peaking at 1 % (Fig. 5C, left panel), while HO-1 protein levels increased dose-dependently (right panel). CD163 and Nrf2 mRNA followed similar expression patterns (Fig. 5D and E). Ferritin heavy chain mRNA was also markedly upregulated, suggesting efficient iron sequestration (Fig. 5F). At higher hematocrits (2.5 %), a suppression of mRNA induction was noted, likely reflecting apoptosis triggered by excessive erythrophagocytosis [48]. Time-course analyses (Supplementary Fig. 6A) revealed that Nrf2 and HO-1/HMOX1 induction preceded ferritin upregulation, consistent with iron-driven activation of the Nrf2/HO-1 axis. Free heme levels measured in the supernatant during erythrophagocytosis remained unchanged (Supplementary Fig. 6B), indicating efficient heme detoxification. Further experiments using GOX (a specific H_2_O_2_ source) confirmed that both heme and H_2_O_2_—but not CuSO_4_—induced HO-1/HMOX1 expression (Supplementary Fig. 6C).

In summary, co-culture of THP-1-derived macrophages with oxidized RBCs provides a robust and mechanistically informative model to study erythrophagocytosis, with HO-1/HMOX1 mRNA serving as a sensitive functional readout.

### 3.7 Ethanol primes RBCs for erythrophagocytosis

**Fig. 6:**
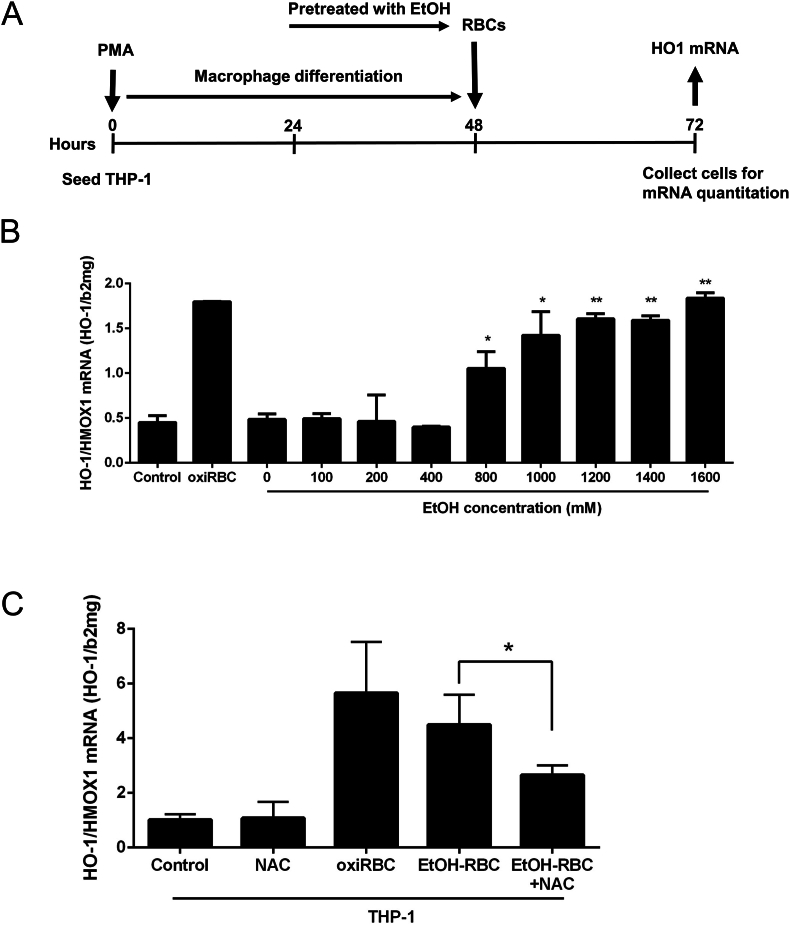
Ethanol primes RBCs for erythrophagocytosis. **A)** Experimental design: RBC were exposed to increasing concentrations of ethanol for 24 h. RBCs were then co-cultured with THP-1 macrophages for 24 h. **B)***HO-1/HMOX1* mRNA is significantly increased starting from 800 mM pre-exposure of RBC to ethanol. Control: THP-1 macrophages alone, oxiRBC: CuSO_4-_oxidized RBC. **C) N-acetylcysteine (NAC) efficiently blocks ethanol-mediated priming of RBC for erythrophagocytosis.** 0.5 % RBCs were pretreated with 800 mM ethanol for 24 h, with or without 2 mM NAC (EtOH-RBC and EtOH-RBC + NAC). RBCs were then co-cultured with THP-1 macrophages. Additional conditions include: untreated THP-1 cells (Control), THP-1 cells treated with NAC alone, and THP-1 cells co-cultured with CuSO_4_-oxidized RBCs (oxiRBC). **The bar graph shows HO-1 (HMOX1) mRNA expression in THP-1 macrophages under these five conditions, as a marker of erythrophagocytosis-induced oxidative stress.** NAC alone has no effect; oxidized RBCs and ethanol-pretreated RBCs strongly induce HO-1; NAC co-treatment reduces this induction, indicating protective effects against ethanol-mediated erythrocyte priming. mRNA was quantified by qRT-PCR in triplicates, normalized to β2-microglobulin, and results are presented as mean ± SD. ∗P < 0.05, ∗∗P < 0.01.

We next examined the effect of a prolonged period of incubation time (24-h) in human control RBCs with varying concentrations of ethanol (Fig. 6A and B). Unlike the direct lysis of RBCs by ethanol (see Fig. 1F), a one-day incubation with ethanol at concentrations as low as 800 mM (∼5 %) primed RBCs for erythrophagocytosis. Remarkably, an ethanol treatment period as short as 2 h was sufficient to prime RBCs for erythrophagocytosis. We also confirmed that ethanol evaporation during the incubation was minimal (<5 %, data not shown). To explore the protective role of the antioxidant N-acetylcysteine (NAC), which is a common treatment for patients with alcoholic hepatitis [58], RBCs were co-incubated with ethanol in the presence or absence of NAC before co-culturing with THP-1-derived macrophages. As shown in Fig. 6C, NAC significantly blocked ethanol-mediated priming of RBCs for erythrophagocytosis.

**Fig. 7:**
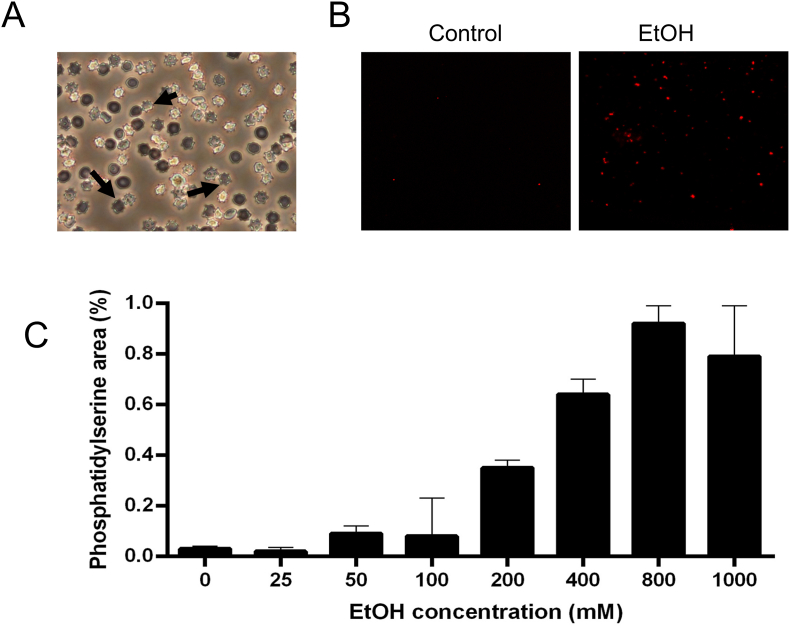
Induction of eryptosis by ethanol. A) Morphological signs of suicidal erythrocyte death (eryptosis) which is characterized by cell shrinkage, cell membrane blebbing, and cell membrane phospholipid scrambling with phosphatidylserine exposure at the cell surface. A representative image was taken using live-cell culture microscopy. Red blood cells (RBCs) were incubated with 800 mM ethanol for 2 h. The image shown is representative and digitally enhanced for clarity. **B)** Phosphatidylserine exposure on RBC membranes following ethanol treatment was assessed using Annexin V staining. Representative images from three independent experiments for each ethanol concentration are shown. Phosphatidylserine was indirectly stained using Annexin V as a primary antibody (Proteintech, #11060-1-AP, dilution 1:500) and an Alexa Fluor 647-conjugated donkey anti-rabbit IgG as a secondary antibody (Dianova, #711605152, dilution 1:500). **C)** Quantification of phosphatidylserine staining intensity. Data are presented as mean values derived from triplicate measurements.

To investigate the underlying mechanisms of ethanol-primed erythrophagocytosis, we measured PS, a phospholipid normally restricted to the inner leaflet of the RBC membrane. During eryptosis—a programmed form of RBC death similar to apoptosis in nucleated cells [18] —PS externalizes to the outer leaflet, serving as a key signal for macrophages to recognize and engulf dying RBCs [18]. Living cell culture light microscopy revealed morphological signs of eryptosis with blebbing and shrinkage (Fig. 7A). Confocal microscopy images of PS in ethanol-treated RBCs are shown Fig. 7B (control versus 800 mM EtOH). RBCs were treated with varying concentrations of ethanol for 2 h. Fig. 7C illustrates the quantitative data with a significant induction of PS externalization, indicating eryptosis during ethanol treatment.

In conclusion, ethanol primes RBCs for erythrophagocytosis, likely due to eryptosis, and this effect can be effectively suppressed by NAC.

### 3.8 Ethanol reversibly blocks erythrophagocytosis at increased concentrations

The in vitro erythrophagocytosis system enabled us to investigate alcohol's potential inhibitory effects on RBC ingestion (experimental design outlined in Suppl. Fig. 7A). Oxidized RBCs (1 %) were co-incubated with THP-1-derived macrophages for 24 h in the presence of varying ethanol concentrations. As shown in Suppl. Fig. 7B, 100 mM ethanol had no effect on erythrophagocytosis, but levels ≥200 mM significantly inhibited the process. According to HMOX1 mRNA expression (Suppl. Fig. 7B) and viability data (Suppl. Fig. 7C), macrophage regeneration was impaired at 200 mM ethanol, while HMOX1 remained inducible. Overt toxicity appeared at concentrations of 400 mM and above. However, macrophages recovered after 24 h and regained erythrophagocytic activity, as indicated by increased *HO-1/HMOX1* mRNA expression upon exposure to oxidized RBCs (Suppl. Fig. 7D). In summary, ethanol exerts a dual effect on erythrophagocytosis. While it primes RBCs for ingestion, prolonged exposure to high ethanol levels reversibly inhibits macrophage phagocytic activity.

### 3.9 Heme-degradation product bilirubin and free heme/hemin also prime RBCs for erythrophagocytosis

**Fig. 8:**
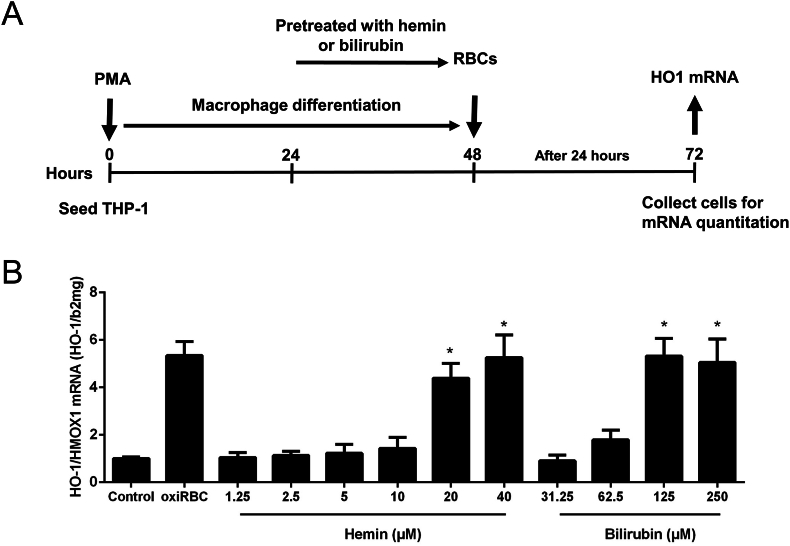
RBC degradation end products heme and bilirubin prime RBCs for erythrophagocytosis. **A)** Experimental design: RBC were exposed to increasing concentrations of hemin or bilirubin for 3 h. RBCs treated with CuSO_4_ for 2 h served as positive control (oxiRBC). RBCs were then co-cultured with THP1 macrophages for 24 h. **B)***HO-1/HMOX1* mRNA are significantly increased starting from 10 μM pre-exposure to hemin or 62.5 μM preexposure to bilirubin. *HO-1/HMOX1* mRNA was quantified by quantitative real-time PCR using triplicates and the results are represented as mean of mRNA levels normalized to β2-microglobulin ±SD. ∗P < 0.05 (vs. control).

Given the toxicity of high levels of heme that accumulate during hemolysis, we investigated their potential to trigger erythrophagocytosis of RBCs from healthy donors. The experimental design is shown in Fig. 8A. Human RBCs were incubated for 24 h with increasing concentrations of bilirubin and hemin at levels typical in end-stage liver disease (e.g., 60 μM bilirubin, Fig. 4C), followed by co-incubation with THP-1-derived macrophages for an additional 24 h. As shown in Fig. 8B, higher bilirubin and hemin concentrations significantly increased *HO-1/HMOX1* mRNA expression, indicating enhanced erythrophagocytosis since increased HO-1 is one indicator of erythrophagocytosis. This effect was observed starting at 10 μM for hemin and 62.5 μM for bilirubin.

In conclusion, hemolysis end-products like bilirubin and free heme/heme can amplify erythrophagocytosis, potentially creating a self-perpetuating cycle in end-stage liver disease.

## 4 Discussion

Our studies provide novel insights into the role of hemolysis and erythrophagocytosis in ALD. These findings may also explain a number of commonly observed laboratory findings in ALD patients such as hepatic iron accumulation or elevation of MCV, anemia or elevated ferritin. First, in human heavy drinkers, we observed direct optical signs of hemolysis, elevated sCD163 levels, and other hemolysis markers, including LDH and ferritin, which improved following alcohol detoxification. The negative correlation between LDH and hemoglobin highlights the hemolytic nature of low hemoglobin levels, both of which strongly associate with long-term mortality, emphasizing the critical role of hemolytic anemia in ALD. sCD163 levels also correlated positively with markers of liver damage and fibrosis stage. Furthermore, hepatic CD163 mRNA expression was associated with iron accumulation and fibrosis and was significantly induced in precision-cut liver slices exposed to ethanol-treated RBCs.

RBCs from heavy drinkers exhibited increased fragility under hemolytic stress, suggesting that ethanol may have a direct damaging effect on RBCs that is independent of folic acid and vitamin B12 deficiency. In a murine model of chronic alcohol exposure, we validated hemolysis through visible optical signs and elevated hepatic CD163 expression. The co-localization of CD163 with RBC autofluorescence directly demonstrated enhanced hepatic erythrophagocytosis associated with exposure to high levels of alcohol. Additionally, we replicated ethanol-mediated erythrophagocytosis in vitro using human RBCs and macrophages. Ethanol primed RBCs due to eryptosis, as evidenced by elevated PS levels which started at ethanol concentrations as low as 50 mM (0.3 %) [18]. Interestingly, ethanol exerted a dual effect: it primed RBCs for ingestion while prolonged exposure to high ethanol concentrations reversibly inhibited macrophage phagocytic activity. While reduced cell viability may contribute to this effect, normalization to cell number suggests a genuine, transient functional impairment. Heme that is released in large quantities during hemolysis may further amplify erythrophagocytosis and may potentially cause a self-perpetuating cycle in end-stage liver disease.

To the best of our knowledge, this is the first study to demonstrate that ethanol exposure causes hemolysis and primes intact human RBCs for erythrophagocytosis through eryptosis. RBC recycling predominantly occurs in the spleen and liver through the reticuloendothelial system and macrophages recycle iron for new RBC production in the bone marrow [23]. The increased fragility and hemolysis in heavy drinkers may be due to direct toxicity of alcohol to hematopoietic cells, disrupting blood cell production and altering bone marrow morphology [16]. Vacuolization in erythroid and megakaryocytic lineages has been observed within a week of heavy alcohol intake and resolves after abstinence [59]. These changes persist even with folic acid supplementation [60]. In our cohort, hemolytic anemia was observed without folic acid or vitamin B12 deficiencies [4]. Findings from the chronic ethanol mouse models confirm alcohol's deleterious effects on hematopoietic stem cells, early myeloid progenitors, and erythroid maturation [13]. However, more detailed studies on the direct toxic effects of alcohol on the bone marrow are required for a better understanding of the underlying mechanisms.

Our in vitro findings suggest that ethanol induces eryptosis, a programmed RBC death characterized by PS externalization, which serves as a macrophage recognition signal for erythrophagocytosis [18]. Eryptosis, akin to apoptosis in nucleated cells, removes defective erythrocytes prior to hemolysis. While this process can mitigate defective RBCs, excessive eryptosis may impair microcirculation and lead to anemia [35]. Interestingly, NAC partially blocked erythrophagocytosis, suggesting it interacts with eryptosis induction rather than directly with the RBC lipid layer. Future studies should investigate ethanol's interference with eryptosis and explore potential therapeutic strategies targeting this process.

The in vitro erythrophagocytosis system also allowed us to examine the priming effects of molecules such as heme and bilirubin. While not directly proven, our findings suggest that these end-products may sustain hemolysis or eryptosis, creating a vicious cycle in patients with liver disease. Notably, bilirubin levels observed in cirrhotic ALD patients matched those required to prime RBCs for erythrophagocytosis. This may also explain the observed increase in sCD163 with advancing fibrosis stages. Although bilirubin has demonstrated antioxidant properties in certain contexts, it is predominantly toxic in biological systems, as exemplified by kernicterus in newborns [61]. Recent studies also show that bilirubin and bile acids can induce eryptosis [62,63]. Taken together, these findings underscore the toxic role of heme degradation products such as bilirubin in inducing eryptosis.

Another consideration is whether the high alcohol concentrations required to prime RBCs for erythrophagocytosis are achievable in vivo. In the patient cohort of heavy drinkers, the mean blood alcohol concentration was 0.1 % (17 mM), but transiently higher levels may occur in distinct compartments such as the gastrointestinal tract or portal venous system during binge drinking [64,65]. Even at 50 mM (0.3 %), alcohol-induced PS elevation suggests a plausible mechanism for increased RBC turnover. This concept aligns with clinical observations that binge drinking elevates liver disease risk independently of average alcohol intake [7,66]. Thus, enhanced hemolysis, eryptosis, and erythrophagocytosis may contribute to the pathogenesis of ALD and alcoholic hepatitis [11]. Of note, we also demonstrated that alcohol can reversibly block erythrophagocytosis, but such levels are not reached in spleen nor livers.

The use of sCD163 as a marker of erythrophagocytosis is supported by its molecular role as an endocytic receptor for hemoglobin-haptoglobin complexes [67]. While widely used as a surrogate marker for liver fibrosis and inflammation [[38], [39], [40]], our findings highlight its specific association with hemolysis. sCD163 levels correlated with fibrosis stages and normalized during alcohol detoxification, supporting its use as a surrogate marker for hemolysis.

Finally, our findings provide insights into the poorly understood iron accumulation in heavy drinkers [4]. Hemolytic markers correlated with ferritin, and *CD1*63 mRNA was associated with hepatocyte iron and ferroptosis markers such as GPX4 [68]. Ferroptosis, an iron-dependent cell death process, may be connected to heme turnover [69]. Future studies should explore the interplay between hemolysis, erythrophagocytosis, and ferroptosis in liver injury.

In conclusion, we here demonstrate that alcohol triggers hemolysis and erythrophagocytosis both in vitro and in vivo. Enhanced erythrophagocytosis, driven by heme and bilirubin, may create a vicious cycle of RBC lysis and liver injury. These findings establish a novel link between alcohol, RBC turnover, and liver disease, offering potential targets for therapeutic intervention. Interventions such as treatment with NAC, which has shown mortality benefits in alcoholic hepatitis [70], may hold promise in mitigating complications of alcohol-related liver damage.

## CRediT authorship contribution statement

**Chaowen Zheng:** Writing – review & editing, Investigation, Formal analysis. **Siyuan Li:** Writing – review & editing. **Johannes Mueller:** Writing – review & editing. **Cheng Chen:** Investigation. **Huanran Lyu:** Writing – review & editing. **Guandou Yuan:** Writing – review & editing, Resources. **Ane Zamalloa:** Investigation. **Lissette Adofina:** Investigation. **Parthi Srinivasan:** Investigation. **Krishna Menon:** Investigation. **Nigel Heaton:** Investigation. **Stephan Immenschuh:** Writing – review & editing. **Ines Silva:** Writing – review & editing, Investigation. **Vanessa Rausch:** Writing – review & editing. **Seddik Hammad:** Resources. **Steven Dooley:** Resources. **Shilpa Chokshi:** Resources. **Antonio Riva:** Writing – review & editing, Resources. **Songqing He:** Writing – review & editing. **Sebastian Mueller:** Writing – review & editing, Writing – original draft, Supervision, Resources, Funding acquisition, Conceptualization.

## Declaration of competing interest

The authors declare that they have no known competing financial interests or personal relationships that could have appeared to influence the work reported in this paper.

## Data Availability

Data will be made available on request.

## References

[1] World Health Organization (2018). Global status report on alcohol and health. http://www.who.int/substance_abuse/publications/global_alcohol_report/en/.2018.

[2] Mueller S., Heilig M. (2023).

[3] Mueller S., Seitz H.K., Rausch V. (2014). Non-invasive diagnosis of alcoholic liver disease. World J. Gastroenterol..

[4] Mueller S., Chen C., Mueller J., Wang S. (2022). Novel insights into alcoholic liver disease: iron overload, iron sensing and hemolysis. J. Transl. Int. Med..

[5] Mueller S., Rausch V. (2015). The role of iron in alcohol-mediated hepatocarcinogenesis. Adv. Exp. Med. Biol..

[6] Silva I., Rausch V., Seitz H.K., Mueller S. (2017). Does hypoxia cause carcinogenic iron accumulation in alcoholic liver disease (ALD)?. Cancers (Basel).

[7] Ventura-Cots M., Watts A.E., Bataller R. (2017). Binge drinking as a risk factor for advanced alcoholic liver disease. Liver Int..

[8] Ding C., Ng Fat L., Britton A., Im P.K., Lin K., Topiwala A. (2023). Binge-pattern alcohol consumption and genetic risk as determinants of alcohol-related liver disease. Nat. Commun..

[9] Ruhl C.E., Everhart J.E. (2005). Joint effects of body weight and alcohol on elevated serum alanine aminotransferase in the United States population. Clin. Gastroenterol. Hepatol. : the official clinical practice journal of the American Gastroenterological Association.

[10] Lau K., Baumeister S.E., Lieb W., Meffert P.J., Lerch M.M., Mayerle J. (2015). The combined effects of alcohol consumption and body mass index on hepatic steatosis in a general population sample of European men and women. Aliment. Pharmacol. Ther..

[11] Louvet A., Mathurin P. (2015). Alcoholic liver disease: mechanisms of injury and targeted treatment. Nat. Rev. Gastroenterol. Hepatol..

[12] Mueller S., Mueller J., Mueller S., Heilig M. (2023). Alcohol and alcohol-related Diseases.

[13] Mueller S., Scheller M., Mueller S., Heilig M. (2023). Alcohol and alcohol-related Diseases.

[14] Douglass C.C., Twomey J.J. (1970). Transient stomatocytosis with hemolysis: a previously unrecognized complication of alcoholism. Ann. Intern. Med..

[15] Homaidan F.R., Kricka L.J., Whitehead T.P. (1984). Morphology of red blood cells in alcoholics. Lancet.

[16] Ballard H.S. (1997). The hematological complications of alcoholism. Alcohol Health Res. World.

[17] Zieve L. (1958). Jaundice, hyperlipemia and hemolytic anemia - herefore unrecognized syndrome associated with alcoholic fatty liver and cirrhosis. Ann. Intern. Med..

[18] Lang F., Lang E., Foller M. (2012). Physiology and pathophysiology of eryptosis. Transfus. Med. Hemotherapy.

[19] Immenschuh S., Baumgart-Vogt E., Mueller S. (2010). Heme oxygenase-1 and iron in liver inflammation: a complex alliance. Curr. Drug Targets.

[20] Tenhunen R., Marver H.S., Schmid R. (1968). The enzymatic conversion of heme to bilirubin by microsomal heme oxygenase. Proc. Natl. Acad. Sci. USA..

[21] Maines M.D. (1997). The heme oxygenase system: a regulator of second messenger gases. Annu. Rev. Pharmacol. Toxicol..

[22] Ryter S.W., Alam J., Choi A.M. (2006). Heme oxygenase-1/carbon monoxide: from basic science to therapeutic applications. Physiol. Rev..

[23] Andrews N.C. (1999). Disorders of iron metabolism. N. Engl. J. Med..

[24] Ganz T. (2013). Systemic iron homeostasis. Physiol. Rev..

[25] Wang J., Pantopoulos K. (2011). Regulation of cellular iron metabolism. Biochem. J..

[26] Kapitulnik J., Maines M.D. (2009). Pleiotropic functions of biliverdin reductase: cellular signaling and generation of cytoprotective and cytotoxic bilirubin. Trends Pharmacol. Sci..

[27] Trakshel G.M., Kutty R.K., Maines M.D. (1986). Purification and characterization of the major constitutive form of testicular heme oxygenase. The noninducible isoform. J. Biol. Chem..

[28] Choi A.M.K., Alam J. (1996). Heme oxygenase-1: function, regulation, and implication of a novel stress-inducible protein in oxidant-induced lung injury. Am. J. Respir. Cell Mol. Biol..

[29] Immenschuh S., Ramadori G. (2000). Gene regulation of heme oxygenase-1 as a therapeutic target. Biochem. Pharmacol..

[30] Lim P.J., Duarte T.L., Arezes J., Garcia-Santos D., Hamdi A., Pasricha S.R. (2019). Nrf2 controls iron homeostasis in haemochromatosis and thalassaemia via Bmp 6 and hepcidin. Nat. Metab..

[31] Paine A., Eiz-Vesper B., Blasczyk R., Immenschuh S. (2010). Signaling to heme oxygenase-1 and its anti-inflammatory therapeutic potential. Biochem. Pharmacol..

[32] Ju C., Mandrekar P. (2015). Macrophages and alcohol-related liver inflammation. Alcohol Res..

[33] Zheng C., Li S., Lyu H., Chen C., Mueller J., Dropmann A. (2024). Direct ingestion of oxidized red blood cells (efferocytosis) by hepatocytes. Hepat Med..

[34] Winn N.C., Volk K.M., Hasty A.H. (2020). Regulation of tissue iron homeostasis: the macrophage "ferrostat". JCI Insight.

[35] Klei T.R., Meinderts S.M., van den Berg T.K., van Bruggen R. (2017). From the cradle to the grave: the role of macrophages in erythropoiesis and erythrophagocytosis. Front. Immunol..

[36] Fabriek B.O., Dijkstra C.D., van den Berg T.K. (2005). The macrophage scavenger receptor CD163. Immunobiology.

[37] Graversen J.H., Madsen M., Moestrup S.K. (2002). CD163: a signal receptor scavenging haptoglobin-hemoglobin complexes from plasma. Int. J. Biochem. Cell Biol..

[38] Kawanaka M., Nishino K., Kawada M., Ishii K., Tanikawa T., Katsumata R. (2023). Soluble CD163 is a predictor of fibrosis and hepatocellular carcinoma development in nonalcoholic steatohepatitis. BMC Gastroenterol..

[39] Moller H.J., Gronbaek H., Schiodt F.V., Holland-Fischer P., Schilsky M., Munoz S. (2007). Soluble CD163 from activated macrophages predicts mortality in acute liver failure. J. Hepatol..

[40] Sherman K.E., Meeds H.L., Rouster S.D., Abdel-Hameed E.A., Hernandez J., Tamargo J. (2021). Soluble CD163 identifies those at risk for increased hepatic inflammation & fibrosis. Open Forum Infect. Dis..

[41] Mueller S. (2020).

[42] Thiele M., Rausch V., Fluhr G., Kjaergaard M., Piecha F., Mueller J. (2018). Controlled attenuation parameter and alcoholic hepatic steatosis: diagnostic accuracy and role of alcohol detoxification. J. Hepatol..

[43] Mueller S., Englert S., Seitz H.K., Badea R.I., Erhardt A., Bozaari B. (2015). Inflammation-adapted liver stiffness values for improved fibrosis staging in patients with hepatitis C virus and alcoholic liver disease. Liver Int..

[44] la M.H.P., Lieber C.S., DeCarli L.M., French S.W., Lindros K.O., Jarvelainen H. (2001). Models of alcoholic liver disease in rodents: a critical evaluation. Alcohol Clin. Exp. Res..

[45] Bertola A., Mathews S., Ki S.H., Wang H., Gao B. (2013). Mouse model of chronic and binge ethanol feeding (the NIAAA model). Nat. Protoc..

[46] Clemens M.R., Remmer H., Waller H.D. (1984). Phenylhydrazine-induced lipid peroxidation of red blood cells in vitro and in vivo: monitoring by the production of volatile hydrocarbons. Biochem. Pharmacol..

[47] Sambrano G.R., Steinberg D. (1995). Recognition of oxidatively damaged and apoptotic cells by an oxidized low density lipoprotein receptor on mouse peritoneal macrophages: role of membrane phosphatidylserine. Proc. Natl. Acad. Sci. USA..

[48] Cambos M., Scorza T. (2011). Robust erythrophagocytosis leads to macrophage apoptosis via a hemin-mediated redox imbalance: role in hemolytic disorders. J. Leukoc. Biol..

[49] Jagatia R., Doornebal E.J., Rastovic U., Harris N., Feyide M., Lyons A.M. (2023). Patient-derived precision cut tissue slices from primary liver cancer as a potential platform for preclinical drug testing. EBioMedicine.

[50] Millonig G., Ganzleben I., Peccerella T., Casanovas G., Brodziak-Jarosz L., Breitkopf-Heinlein K. (2012). Sustained submicromolar H2O2 levels induce hepcidin via signal transducer and activator of transcription 3 (STAT3). J. Biol. Chem..

[51] Livak K.J., Schmittgen T.D. (2001). Analysis of relative gene expression data using real-time quantitative PCR and the 2(-Delta Delta C(T)) method. Methods (San Diego, Calif).

[52] Kumar P., Nagarajan A., Uchil P.D. (2018). Analysis of cell viability by the MTT assay. Cold Spring Harb. Protoc..

[53] Mosmann T. (1983). Rapid colorimetric assay for cellular growth and survival: application to proliferation and cytotoxicity assays. J. Immunol. Methods.

[54] Mueller S., Nahon P., Rausch V., Peccerella T., Silva I., Yagmur E. (2017). Caspase‐cleaved keratin‐18 fragments increase during alcohol withdrawal and predict liver‐related death in patients with alcoholic liver disease. Hepatology.

[55] Chen C., Wang S., Yu L., Mueller J., Fortunato F., Rausch V. (2021). H(2)O(2)-mediated autophagy during ethanol metabolism. Redox Biol..

[56] Silva I., Peccerella T., Mueller S., Rausch V. (2019). IL-1 beta-mediated macrophage-hepatocyte crosstalk upregulates hepcidin under physiological low oxygen levels. Redox Biol..

[57] Silva I., Rausch V., Peccerella T., Millonig G., Seitz H.K., Mueller S. (2018). Hypoxia enhances H2O2-mediated upregulation of hepcidin: evidence for NOX4-mediated iron regulation. Redox Biol..

[58] Mueller S., Mueller S., Heilig M. (2023). Alcohol and alcohol-related Diseases.

[59] Ballard H.S. (1980). Alcohol-associated pancytopenia with hypocellular bone marrow. Am. J. Clin. Pathol..

[60] Lindenbaum J., Lieber C.S. (1969). Hematologic effects of alcohol in man in the absence of nutritional deficiency. N. Engl. J. Med..

[61] Hansen T.W. (2001). Bilirubin brain toxicity. J. Perinatol. : Off. J California Perinatal Ass..

[62] Lang E., Gatidis S., Freise N.F., Bock H., Kubitz R., Lauermann C. (2015). Conjugated bilirubin triggers anemia by inducing erythrocyte death. Hepatology.

[63] Lang E., Pozdeev V.I., Gatidis S., Qadri S.M., Haussinger D., Kubitz R. (2016). Bile acid-induced suicidal erythrocyte death. Cell. Physiol. Biochem..

[64] Falconer B., Gladnikoff H. (1934).

[65] Beck I.T., Paloschi G.B., Dinda P.K., Beck M. (1974). Effect of intragastric administration of alcohol on the ethanol concentrations and osmolality of pancreatic juice, bile, and portal and peripheral blood. Gastroenterology.

[66] Aberg F., Helenius-Hietala J., Puukka P., Jula A. (2017). Binge drinking and the risk of liver events: a population-based cohort study. Liver Int..

[67] Kristiansen M., Graversen J.H., Jacobsen C., Sonne O., Hoffman H.J., Law S.K. (2001). Identification of the haemoglobin scavenger receptor. Nature.

[68] Dixon S.J., Lemberg K.M., Lamprecht M.R., Skouta R., Zaitsev E.M., Gleason C.E. (2012). Ferroptosis: an iron-dependent form of nonapoptotic cell death. Cell.

[69] Nishizawa H., Yamanaka M., Igarashi K. (2022). Ferroptosis: regulation by competition between NRF2 and BACH1 and propagation of the death signal. FEBS J..

[70] Nguyen-Khac E., Thevenot T., Piquet M.A., Benferhat S., Goria O., Chatelain D. (2011). Glucocorticoids plus N-acetylcysteine in severe alcoholic hepatitis. N. Engl. J. Med..

