# Supplementary material for: Evidence for alcohol-mediated hemolysis and erythrophagocytosis
Source: Redox Biol. 2025 Jun 26;85:103742. doi: 10.1016/j.redox.2025.103742 (PMC12268204; doi:10.1016/j.redox.2025.103742)
Supplement: Multimedia component 1 [file mmc1.docx]

***Supplementary Material***

**Supplemental tables**

**Suppl. Tab. 1 Patient characteristics**

| **Patient characteristics (N=1121)** |  |  |  |
| --- | --- | --- | --- |
| **Parameter** | **Mean +/- SD** | **Normal range**  **female** | **Normal range**  **male** |
| **General^a^** |  |  |  |
| Gender (1: male) | 70% |  |  |
| Age (years) | 52.3 +/- 11.5 |  |  |
| BMI (kg/m^2^) | 25.5 +/- 4.7 | 18.5-25 | 18.5-25 |
| Alcohol consumption (g/day) | 185 +/- 120 | <20 | <40 |
| Duration of heavy alcohol drinking (years) | 13.6 +/- 9.9 | 0 | 0 |
| **Laboratory^b^** |  |  |  |
| AST (U/L) | 94 +/- 101 | <35 | <50 |
| ALT (U/L) | 65 +/- 79 | <35 | <50 |
| GGT (U/L) | 410 +/- 662 | <40 | <60 |
| AP (U/L) | 111 +/- 75 | 35-104 | 40-129 |
| Bilirubin total (mg/dL) | 1.53 +/- 3.28 | <1.2 | <1.2 |
| Bilirubin indirect (mg/dL) | 0.47 +/- 0.78 | <0.9 | <0.9 |
| Hemoglobin (g/dL) | 14.0 +/- 2.0 | 12-16 | 13.5-17.5 |
| Ferritin (ng/mL) | 600 +/- 661 | 15-150 | 30-400 |
| Cholesterol (mg/dL) | 214 +/- 68 | <200 | <200 |
| LDH (U/L) | 235 +/- 112 | <250 | <250 |
| sCD163 before detox (ng/mL)^d^ | 1543 +/- 1003 | <800 | <800 |
| sCD163 after detox (ng/mL) | 214 +/- 264 | <800 | <800 |
| **Ultrasound and TE^c^** |  |  |  |
| Liver size (cm) | 16.1 +/- 2.7 | <16 | <16 |
| Hepatic steatosis (US) (0-3) | 1.83 +/- 0.91 | 0 | 0 |
| Spleen size (cm) | 10.2 +/- 2.2 | <11.5 | <11.5 |
| Ascites (1 or 0) | 10% | 0 | 0 |
| Signs of cirrhosis (US) (1 or 0) | 19% | 0 | 0 |
| Liver stiffness (kPa) | 17.8 +/- 22.3 | <6 | <6 |
| CAP (dB/m) | 289 +/- 57 | <236 | <236 |

^a^ reference values from the World Health Organization (WHO), (of note, no specific daily consumption of alcohol is safe)
^b^ reference values from https://www.labor-limbach.de/
^c^ reference values from European Federation of Societies for Ultrasound in Medicine and Biology (EFSUMB)

^d^ Normal range for cCD163 were derived from Azuma Y et al Front Pediatr. 2020;8:148., and Moller HJ et al J Hepatol. 2007;47(5):671-6.

**Suppl. Tab. 2 Mice characteristics**

| **Mouse characteristics** | | | | |  |
| --- | --- | --- | --- | --- | --- |
|  | **Controls** | | **Chronic EtOH model** | |  |
| **Parameter** | **Mean** | **SD** | **Mean** | **SD** | **P** |
| **Liver weight (g)** | 1.063 | 0.141 | 0.922 | 0.091 | 0.088 |
| **Spleen weight (g)** | 0.046 | 0.011 | 0.043 | 0.008 | 0.602 |
| **Leukocytes** | 4.3 | 2.0 | 2.6 | 1.4 | 0.154 |
| **Erythrocytes** | 9.2 | 0.9 | 9.7 | 0.6 | 0.330 |
| **Hemoglobin (g/dL)** | 15.4 | 1.1 | 16.4 | 1.4 | 0.227 |
| **Hematocrit (%)** | 45.1 | 3.5 | 47.4 | 3.7 | 0.327 |
| **LDH (U/L)** | 424.3 | 363.1 | 1045.0 | 791.1 | 0.118 |
| **AST (U/L)** | 193.0 | 133.4 | 583.8 | 459.9 | 0.076 |
| **ALT (U/L)** | 38.8 | 13.4 | 127.2 | 67.8 | 0.012 |
| **Serum iron (µmol/L)** | 37.0 | 28.0 | 20.8 | 6.0 | 0.239 |
| **Albumin (g/L)** | 24.5 | 12.7 | 23.7 | 6.1 | 0.900 |

**Suppl. Tab. 3 A) Primer list and B) antibody list**

**A**

| **Gene** |  | **Sequence** |
| --- | --- | --- |
| **Human** |  |  |
|  | **Forward** | **5'-CCA GGC AGA GAA TGC TGA GTT C-3'** |
| **HO-1/HMOX1** | **Reverse** | **5'- AAG ACT GGG CTC TCC TTG TTG C-3'** |
|  | **Forward** | **5’-TGA CTT TGT CAC AGC CAA AGA TA-3'** |
| **b2mg** | **Reverse** | **5’-AAT CCA AAT GCG GCA TCT TC-3'** |
| **Nrf2** | **Forward** | **5'-CAC ATC CAG TCA GAA ACC AGT GG-3'** |
|  | **Reverse** | **5'-GGA ATG TCT GCG CCA AAA GCT G-3'** |
| **CD163** | **Forward** | **5'-CCA GAA GGA ACT TGT AGC CAC AG-3'** |
|  | **Reverse** | **5'-CAA GAC ATT CTT TCC AGT TAA TG-3'** |
| **Ferritin HC** | **Forward** | **5'-TGA AGC TGC AGA ACC AAC GAG G-3'** |
|  | **Reverse** | **5'-GCA CAC TCC ATT CAT TCA GCC-3'** |
| **Mouse** |  |  |
| **HO-1/hmox1** | **Forward** | **5'-AGG CTA AGA CCG CCT TCC T-3'** |
|  | **Reverse** | **5'-TGT GTT CCT CTG TCA GCA TCA-3'** |
|  | **Probe** | **UPL #22** |

**B**

| **Antigen** | **Host species** | **Dilution** | **Stock concentration (mg/mL)** | **Company** | **Cat. No.** |
| --- | --- | --- | --- | --- | --- |
| **HO-1** | rabbit | 1:1000 | 1.0 | Enzo | ADI-SPA-896 |
| **β-actin** | mouse | 1:3000 | 2.0-2.5 | Sigma | A1978 |
| **CD163** | rabbit | 1:500 | 0.5 | Biorbyt | orb13303 |
| **CD163** | human | 1:500 | 0.5 | Biorbyt | orb390857 |
| **CD163** | mouse | 1:500 | 0.638-0.771 | Abcam | EPR19518 |
| **Rabbit Ig** | goat-HRP conj | 1:3000 | 2.0 | Rockland | 611-1322 |
| **Mouse Ig** | goat-HRP conj | 1:3000 | 2.0 | Abcam | ab6789-1 |
| **Rabbit IgG** | Donkey Alexa Fluor® 647-conjugated | 1:500 |  | jacksonimmuno.com | 711-605-152 |

|  |  |  |  |  |  |
| --- | --- | --- | --- | --- | --- |

**Suppl. Tab. 4:** **Correlation analysis of important hemolytic serum markers A) LDH, hemoglobin and B) sCD163.** Spearman correlations were performed for LDH and hemoglobin on n=1024 ALD patients and for sCD163 on sera of n=229 ALD patients.

**Suppl. Tab. 4A**

| **Spearman** | **LDH** |  | **Hemoglobin** |  |
| --- | --- | --- | --- | --- |
|  | **r** | **p** | **r** | **p** |
| **LDH** |  |  | -0.137 | 3.3E-04 |
| **Hemoglobin** | -0.137 | 3.3E-04 |  |  |
| **AST** | 0.614 | 2.7E-72 | -0.081 | 5.5E-03 |
| **ALT** | 0.429 | 4.0E-32 | 0.156 | 8.9E-08 |
| **GGT** | 0.381 | 5.1E-25 | -0.106 | 3.2E-04 |
| **AP** | 0.354 | 1.3E-21 | -0.286 | 2.8E-23 |
| **Bilirubin total** | 0.371 | 1.0E-23 | -0.225 | 1.0E-14 |
| **Bilirubin indirect** | 0.294 | 2.2E-06 | -0.127 | 3.3E-02 |
| **Quick** | -0.222 | 4.8E-09 | 0.380 | 7.3E-41 |
| **INR** | 0.186 | 9.1E-07 | -0.378 | 1.3E-40 |
| **Urea** | -0.111 | 3.6E-03 | -0.095 | 1.2E-03 |
| **Uric acid** | 0.004 | 9.5E-01 | -0.011 | 8.8E-01 |
| **Creatinine** | -0.117 | 2.2E-03 | 0.050 | 8.9E-02 |
| **Lipase** | 0.225 | 4.1E-09 | -0.093 | 2.0E-03 |
| **PTT** | 0.162 | 6.3E-05 | -0.267 | 7.7E-18 |
| **Hematocrit** | -0.182 | 1.6E-06 | 0.959 | 0.0E+00 |
| **Erythrocytes** | -0.257 | 7.7E-12 | 0.847 | 1.4E-320 |
| **Leucocytes** | -0.042 | 2.7E-01 | 0.060 | 3.9E-02 |
| **Ferritin** | 0.372 | 2.5E-23 | 0.061 | 3.9E-02 |
| **CRP** | 0.196 | 2.2E-07 | -0.313 | 8.7E-28 |
| **Albumin** | -0.227 | 4.0E-07 | 0.473 | 5.0E-50 |
| **Protein total** | -0.055 | 1.8E-01 | 0.351 | 2.4E-30 |
| **Transferrin** | -0.249 | 1.3E-07 | 0.325 | 7.5E-22 |
| **Transferrin saturation** | 0.182 | 1.6E-04 | 0.023 | 5.2E-01 |
| **Serum iron** | 0.045 | 2.8E-01 | 0.263 | 3.5E-17 |
| **Triglycerides** | -0.042 | 3.1E-01 | 0.220 | 1.9E-12 |
| **Cholesterol** | 0.060 | 1.4E-01 | 0.264 | 1.7E-17 |
| **Bile acids** | 0.710 | 4.4E-03 | -0.130 | 3.1E-01 |
| **Haptoglobin** | -0.283 | 7.6E-06 | 0.242 | 4.0E-08 |
| **CD163** | 0.454 | 1.2E-09 | -0.296 | 5.2E-06 |
| **M30** | 0.424 | 6.7E-16 | -0.153 | 1.0E-04 |
| **APO A1** | 0.001 | 9.8E-01 | 0.274 | 9.4E-09 |
| **APO A1 after detox** | -0.206 | 1.7E-01 | 0.133 | 1.9E-01 |
| **Hemopexin** | -0.128 | 3.8E-01 | 0.203 | 8.9E-02 |
| **Liver size** | 0.115 | 5.7E-03 | -0.030 | 3.5E-01 |
| **Hepatic steatosis** | 0.163 | 2.0E-04 | -0.055 | 1.1E-01 |
| **Spleen size** | 0.049 | 2.5E-01 | -0.128 | 7.5E-05 |
| **Ascites** | 0.162 | 4.4E-05 | -0.372 | 3.9E-37 |
| **Sings of cirrhosis** | 0.218 | 3.8E-08 | -0.373 | 1.1E-36 |
| **Liver stiffness** | 0.296 | 5.3E-15 | -0.310 | 2.4E-26 |
| **Status death** | 0.197 | 3.6E-05 | -0.232 | 5.3E-11 |

**Suppl. Tab. 4B**

| **Spearman correlations of serum sCD163 (N=229)** | | | | | |
| --- | --- | --- | --- | --- | --- |
| **positive Spearman Rho** | | | **negative Spearman Rho** | | |
| **Parameter** | **r** | **P** | **Parameter** | **r** | **P** |
| Bile acids (µmol/L) | 0.777 | 1.6E-07 | APO A1 after detox (mg/dL) | -0.751 | 2.6E-06 |
| Liver stiffness (kPa) | 0.683 | 1.7E-32 | APO A1 (mg/dL) | -0.643 | 4.1E-13 |
| Bilirubin indirect (mg/dL) | 0.622 | 6.6E-07 | Albumin (g/L) | -0.525 | 2.4E-13 |
| Maddrey score | 0.589 | 1.9E-22 | Transferrin (g/L) | -0.460 | 9.4E-11 |
| Bilirubin total (mg/dL) | 0.584 | 3.8E-22 | Erythrocytes (/pL) | -0.408 | 1.4E-10 |
| M30 (U/L) | 0.544 | 9.0E-19 | Hemoglobin (g/dL) | -0.296 | 5.2E-06 |
| AST (U/L) | 0.504 | 3.6E-16 | Haptoglobin (g/L) | -0.282 | 2.6E-03 |
| GGT (U/L) | 0.503 | 8.0E-16 | Hematocrit (%) | -0.271 | 3.3E-05 |
| LDH (U/L) | 0.454 | 1.2E-09 | Hemopexin (mg/mL) | -0.266 | 2.5E-02 |
| MCV (fL) | 0.376 | 1.3E-08 |  |  |  |
| CRP (mg/dL) | 0.350 | 6.2E-08 |  |  |  |
| Ferritin (ng/mL) | 0.280 | 1.7E-05 |  |  |  |
| ALT (U/L) | 0.215 | 1.1E-03 |  |  |  |

**Suppl. Tab. 5:** **Correlation of hepatic *CD163* mRNA.** Spearman correlations of CD163 mRNA from 30 livers of heavy drinkers.

| **Spearman** | **Category** | **CD163/b2mg (mRNA)** | |
| --- | --- | --- | --- |
|  |  | **r** | **p** |
| **TLR4/b2mg** | mRNA | 0.635 | 0.0002 |
| **PRX2 ox/β-actin** | protein | -0.867 | 0.0003 |
| **Duration of alcohol consumption (years)** | general | 0.602 | 0.0024 |
| **Iron hepatocytes (0-3)** | histology | 0.500 | 0.0129 |
| **Nrf2/b2mg** | mRNA | 0.496 | 0.0162 |
| **PRX2 red/β-actin** | protein | -0.671 | 0.0168 |
| **ALT (U(L)** | laboratory | -0.433 | 0.0169 |
| **Fibrosis stage (0-4)** | elastography | 0.446 | 0.0174 |
| **AST (U/L)** | laboratory | -0.429 | 0.0180 |
| **HbA1C (%)** | laboratory | -0.562 | 0.0189 |
| **GPX4/β-actin** | protein | -0.786 | 0.0208 |
| **LDH (U/L)** | laboratory | -0.447 | 0.0250 |
| **Microgranulomas (0-1)** | histology | 0.391 | 0.0586 |
| **GGT (U/L)** | laboratory | -0.343 | 0.0633 |
| **Hyaluronan (ng/mL)** | special | -0.633 | 0.0671 |
| **Hepcidin (ng/mL)** | special | 0.395 | 0.0690 |
| **Liver stiffness (kPa)** | elastography | 0.331 | 0.0853 |
| **Iron-macrophage (0-3)** | histology | 0.352 | 0.0918 |
| **Bilirubin total (mg/dL)** | laboratory | 0.311 | 0.0947 |
| **Alcohol consumption (g/day)** | general | -0.330 | 0.1150 |
| **Age (years)** | general | 0.293 | 0.1234 |
| **HO-1/b2mg** | mRNA | 0.299 | 0.1651 |

**Supplemental figures**

**
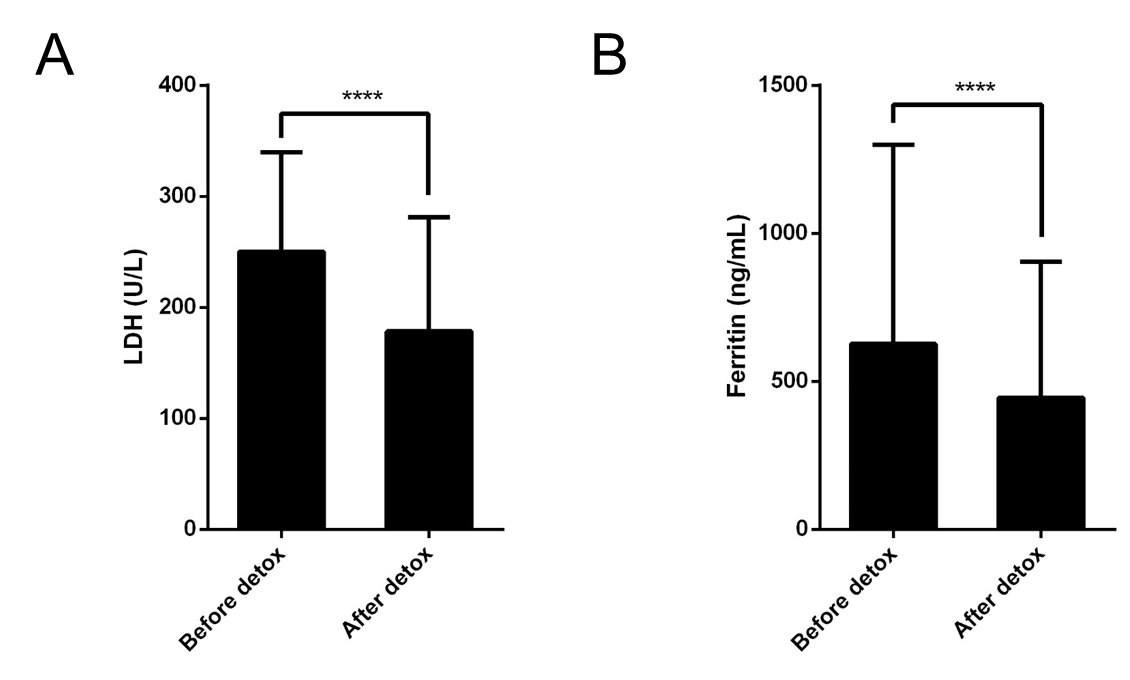
**

**Suppl. Fig. 1: Serum LDH in n=45 and ferritin in n=600 heavy drinkers before and after one week of alcohol detoxification.** ****P<0.0001

**
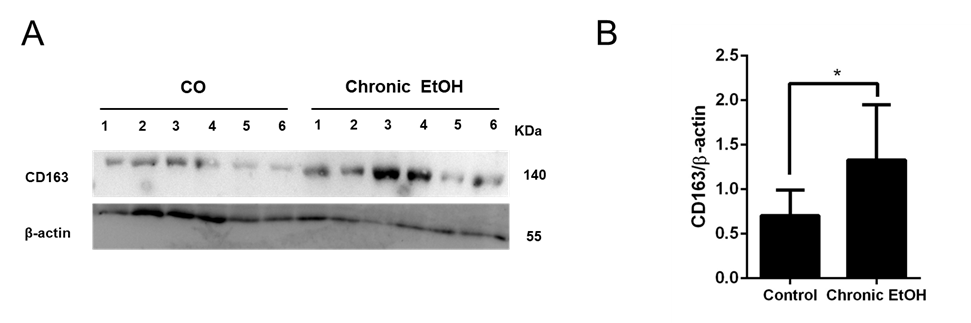
**


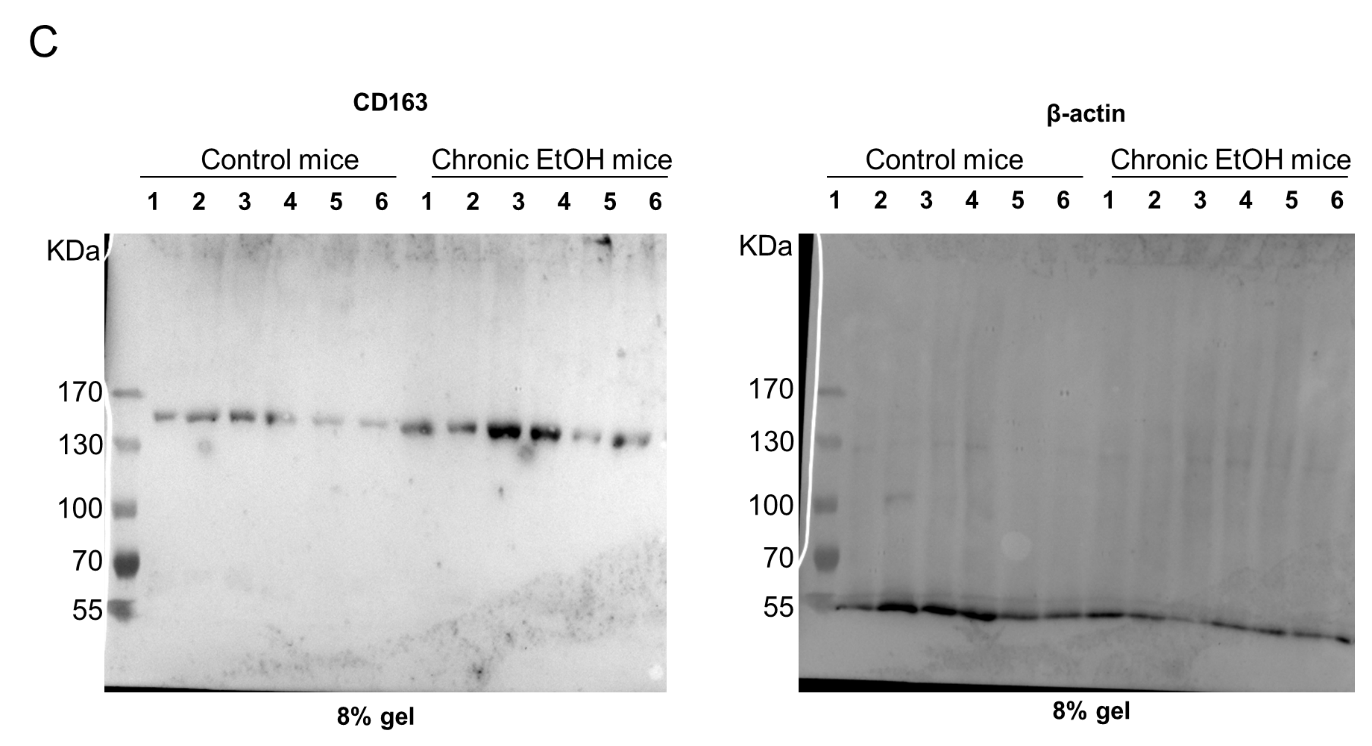


**Suppl. Fig. 2: CD163 and HO-1 are induced in the chronic ALD mice Liver.** Mice were a modified pair-fed Lieber-DeCarli diet for three weeks, followed by a binge phase. **A)** CD163 western blot and **B)** quantification of CD163 protein expression in western blotting. CD163 expression was significantly higher in the chronic alcohol group as compared to the control group. HO-1 is also induced as reported recently by us (for details see suppl. Fig. 1A in: Chen C, Wang S, Yu L, Mueller J, Fortunato F, Rausch V, et al. H(2)O(2)-mediated autophagy during ethanol metabolism. Redox Biol. 2021;46:102081). *P<0.05. **C)** Full Westernblot images for CD163 and Beta-actin.


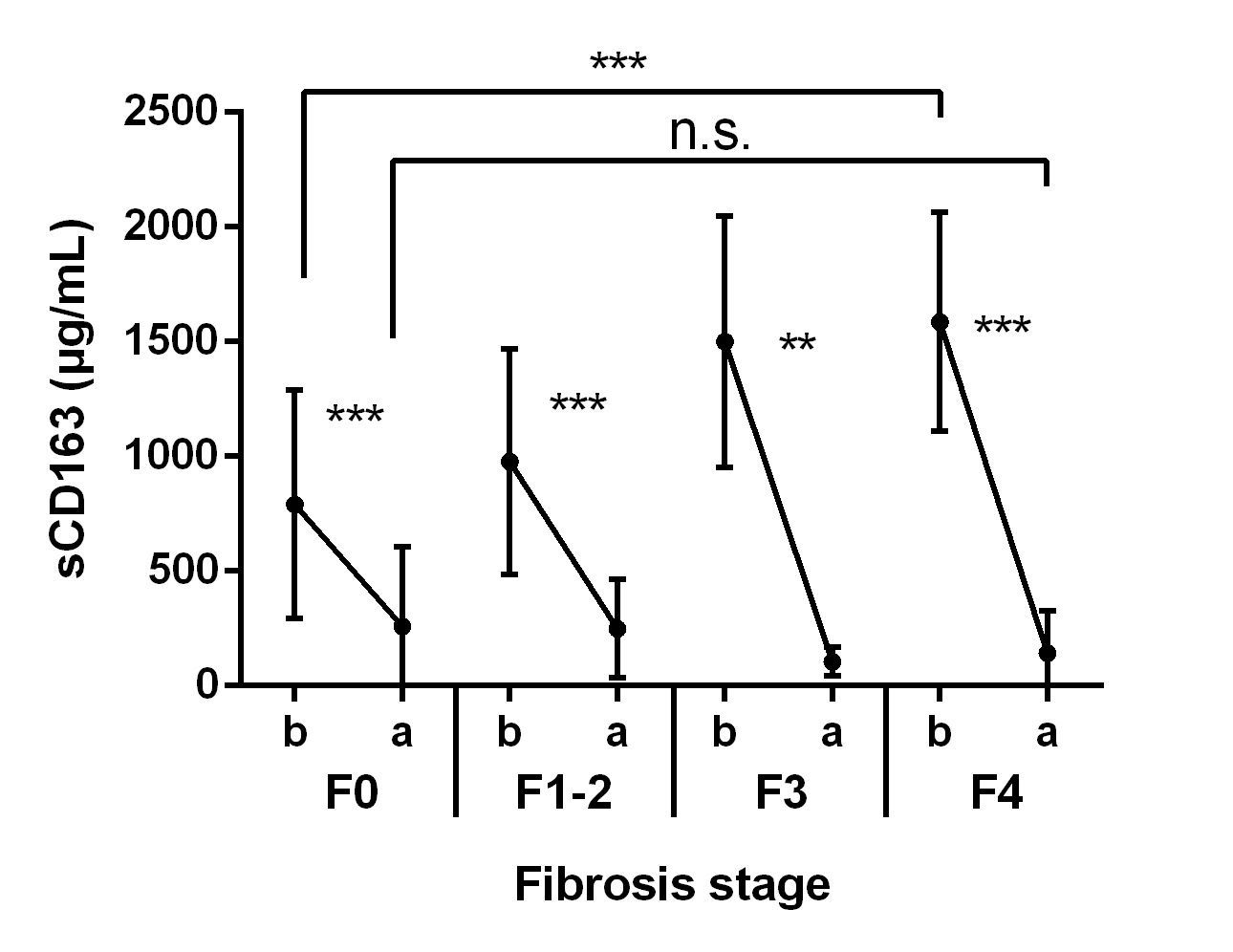


**Suppl. Fig. 3: CD163 serum levels in 67 ALD patients with measurements before (b) and after (a) alcohol detoxification.** Individual numbers were: F0: N=26, F1-2: N=20, F3: N=6 and F4: N=15. **P<0.01, ***P<0.001


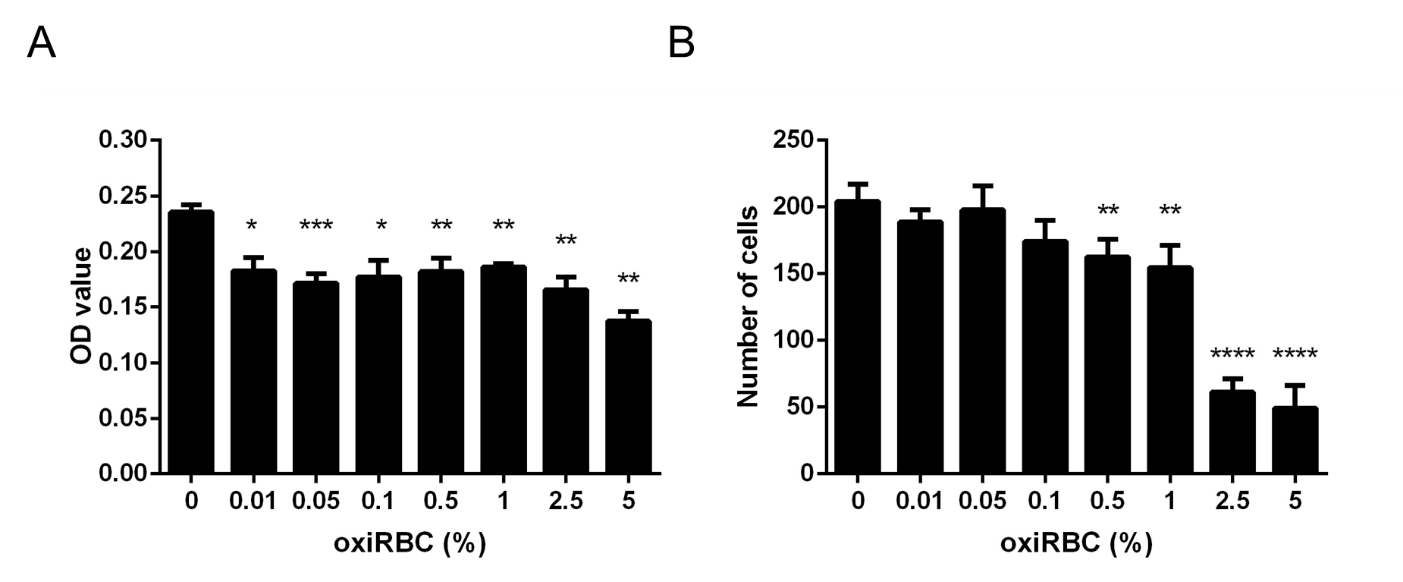


**Suppl. Fig. 4: Cell viability in THP1-derived macrophages after erythrophagocytosis of oxidized RBCs over 24 hours**. Viability was measured using **A)** MTT test (left, n=3 per concentration) and **B) direct** cell counting (right). Cell count was determined by counting cells in 6 independent images per well. Shown is mean and SD. Note that no significant cell death is observed till an oxidized RBC (oxiRBC) concentration of 2.5%. *P<0.05, **p<0.01, ***P<0.001, ****P<0.0001 (Dunnett`s method)


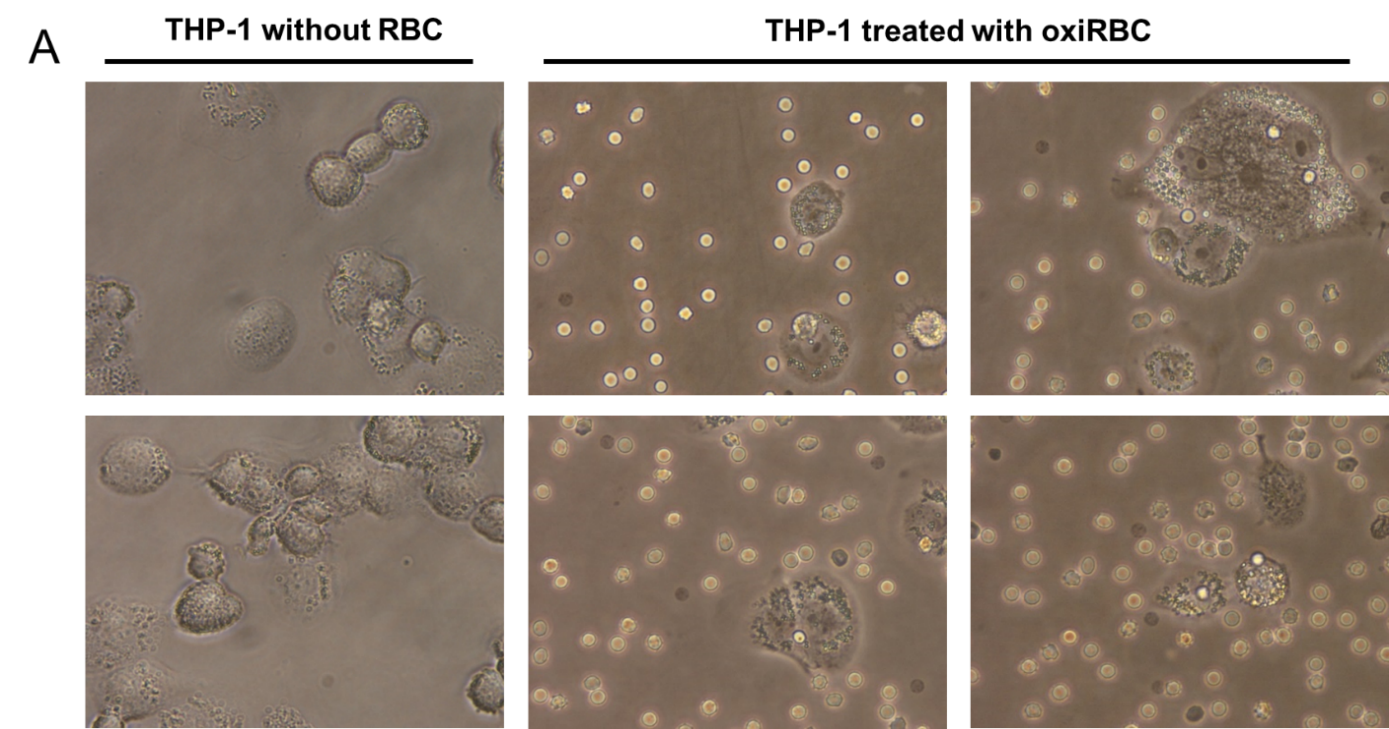


**Suppl. Fig. 5: A) Examples of *in vitro* erythrophagocytosis of oxidized human erythrocytes by THP1-derived macrophages.** This figure demonstrates *in vitro* erythrophagocytosis of oxidized human erythrocytes by THP-1-derived macrophages. For details of the experimental design, refer to Fig. 4. Briefly, THP-1 monocytes were differentiated into macrophages using PMA treatment and subsequently co-cultured with isolated oxidized human RBCs. Notably, in contrast to control THP-1 cells, multiple vacuoles can be observed in THP-1-derived macrophages, indicating active phagocytosis. Additionally, some THP-1-derived macrophages appear enlarged. Morphological changes in certain RBCs, such as the appearance of spur cells or acanthocytes, are evident, which are indicative of eryptosis. Of note, in contrast to hepatocyte-mediated erythrophagocytosis (33), RBCs in macrophages were continuously digested rather than accumulating.


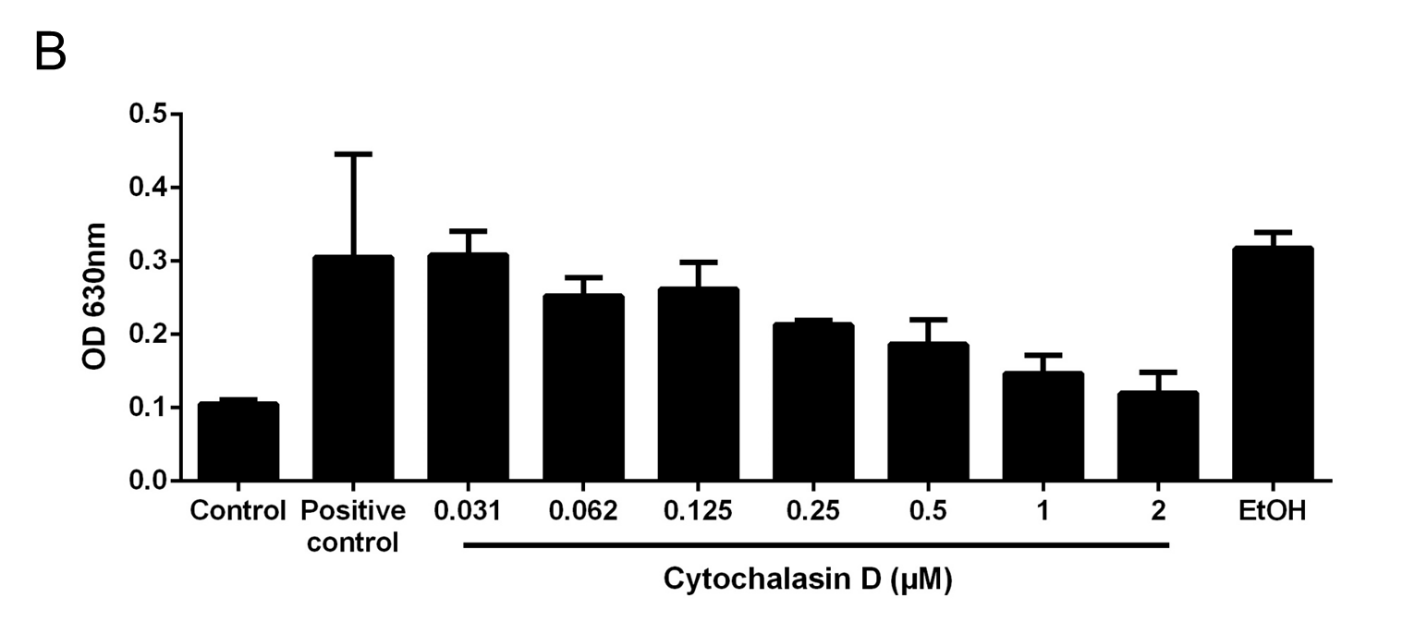


**Supplementary Fig. 5B: Inhibition of Erythrophagocytosis by Cytochalasin D**THP-1 cells were differentiated into macrophages and treated with increasing concentrations of cytochalasin D, followed by incubation with either 1% oxidized RBCs (oxiRBCs) or ethanol-treated RBCs (EtOH-RBCs) for 24 hours. Phagocytosis was quantified using a commercial phagocytosis assay kit (MyBiosource, MBS168645) according to the manufacturer’s instructions, and absorbance was measured at 630 nm using a microplate reader. Cytochalasin D inhibited the phagocytosis of oxiRBCs by THP-1-derived macrophages in a dose-dependent manner. Controls consisted of cells treated with non-oxidized RBCs; the positive control included cells incubated with oxiRBCs without cytochalasin D; EtOH-RBCs were prepared by pretreatment with 800 mM ethanol for 24 hours.


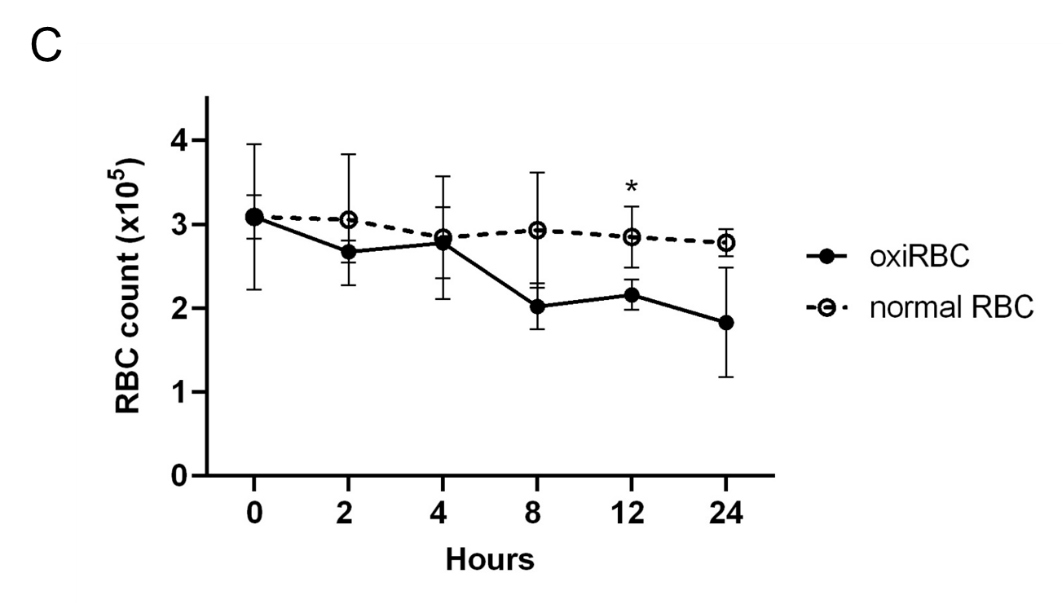


**Suppl. Fig. 5C:** **Continuous elimination of RBCs from the surrounding medium during erythrophagocytosis.** THP-1 monocytes were seeded in 12-well plates at a density of **0.3 × 10⁵ cells/well** and differentiated into macrophages using **PMA**. After 24 hours, **native or oxidized RBCs** were added to the co-culture system. Oxidized RBCs were generated by treating cells with **0.2 mM CuSO₄ and 5 mM ascorbate** for 2 hours, followed by washing with **0.9% sodium chloride** to remove residual oxidants. RBCs were quantified at **2, 4, 8, 12, and 24 hours** using an automated cell counter, with measurements taken **five minutes after co-culture initiation** serving as the baseline.

**
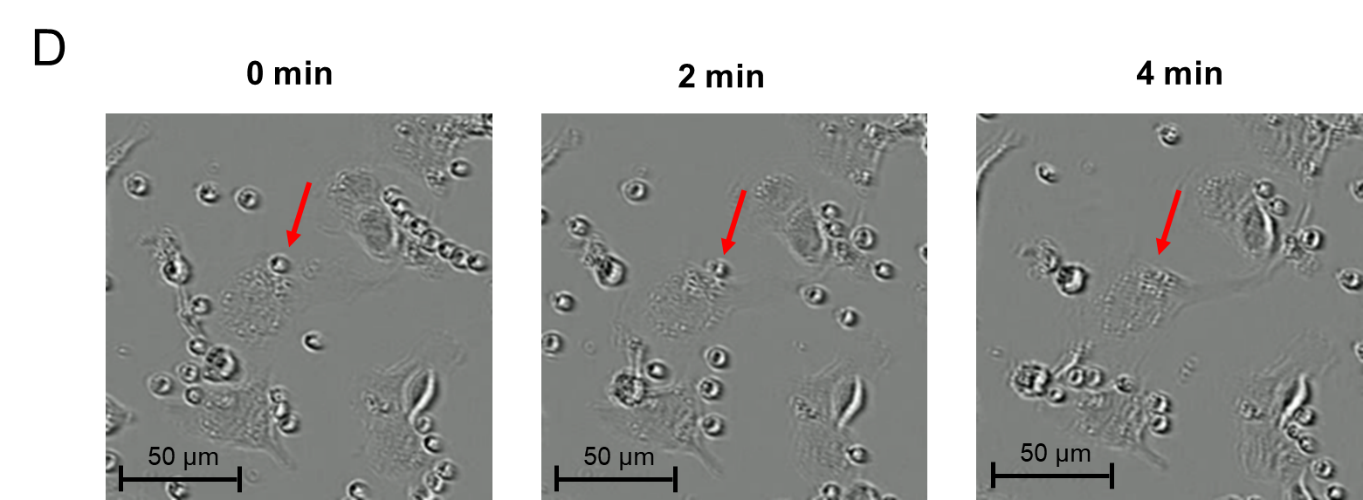
**

**Suppl. Fig. 5D: Rapid Erythrophagocytosis of RBCs by THP-1-Derived Macrophages**THP-1-derived macrophages were co-cultured with 0.05% RBCs pretreated with 800 mM ethanol and monitored by light microscopy using a live-cell video imaging system. Shown is a sequence of representative images capturing the uptake of RBCs at after 0, 2 and 4 minutes. Note, that direct visualization of engulfment events remained infrequent due to temporal resolution limits. Scale bars, 50 µm.


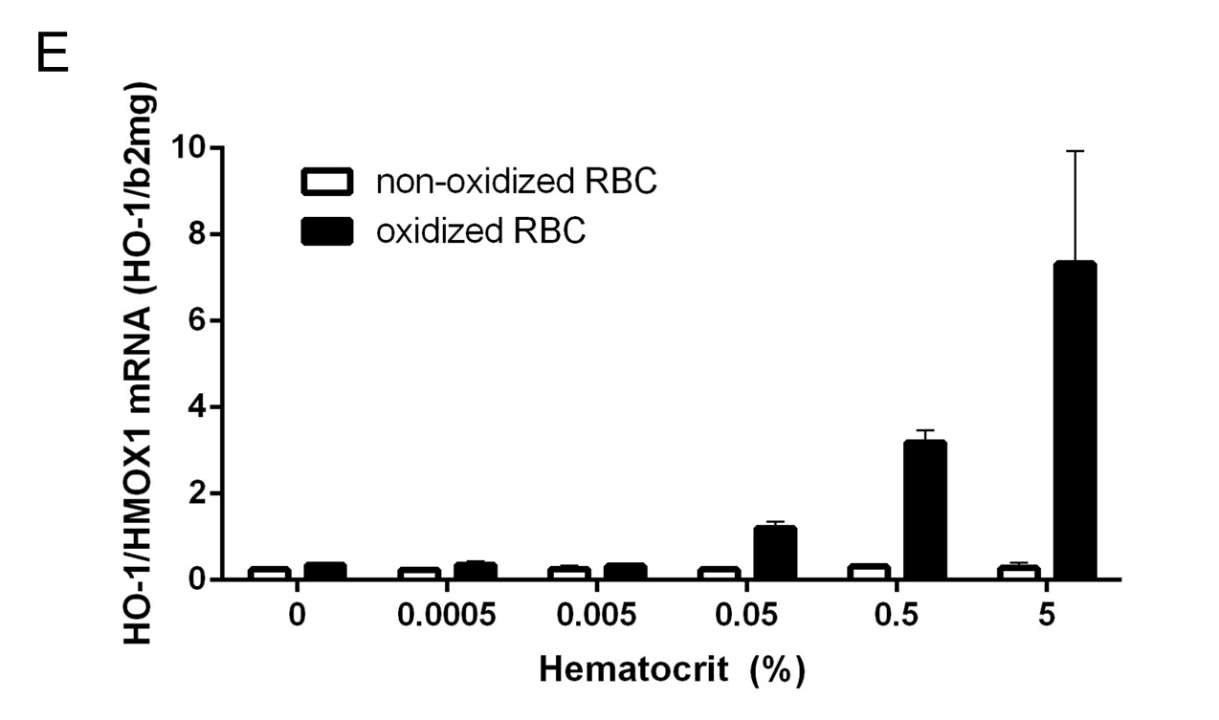


**Suppl. Fig. 5E: In vitro erythrophagocytosis of oxidized human RBCs in primary human macrophages.** Differentiation of primary isolated human monocytes into monocyte-derived macrophages (MDM) for 7 days and exposure to CuSO_4_ treated (oxidized) RBCs also causes a significant induction of *HO-1/HMOX1* mRNA but not in control macrophages. Shown is mean and SD. n=3


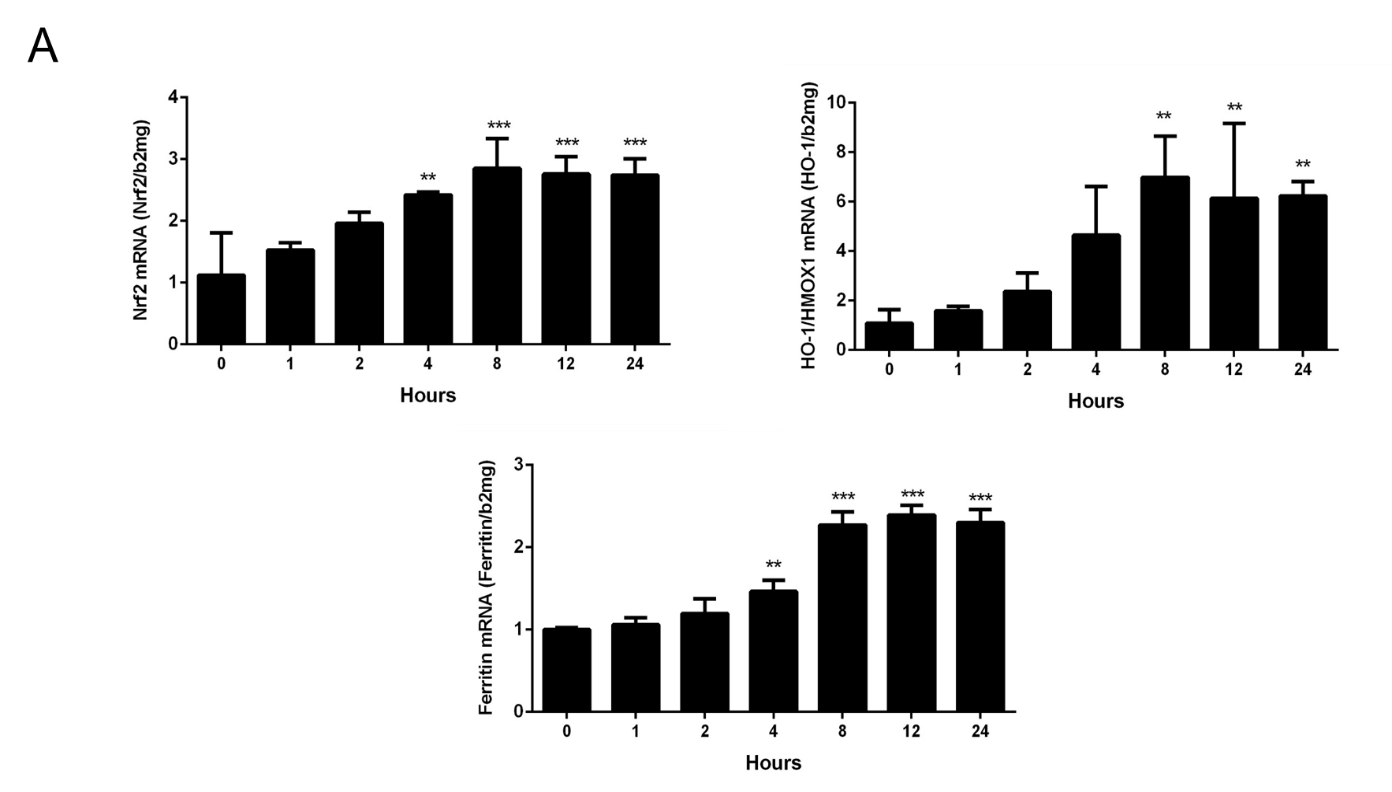


**
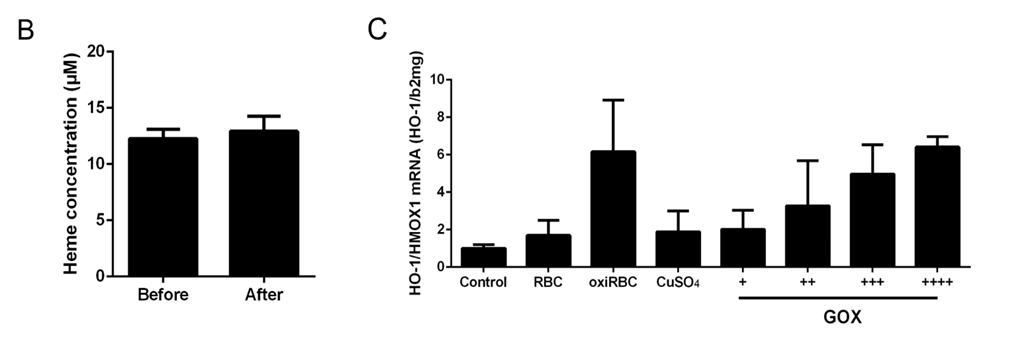
**

**Suppl. Fig. 6: A) Kinetics of Nrf2, HO-1, and Ferritin mRNA Expression During *In Vitro* Erythrophagocytosis of Oxidized Human Erythrocytes.** This figure demonstrates the temporal relationship between *Nrf2*, *HO-1/HMOX1*, and ferritin mRNA expression, indicating that *Nrf2* and *HO-1/HMOX1* activation precedes the upregulation of the iron-storage protein ferritin. This suggests that iron is the primary stimulus for the *Nrf2-HO-1* cascade. The experimental design was similar to that described in Fig. 4. Briefly, THP-1 monocytes were differentiated into macrophages through PMA treatment over 24 hours. The THP-1 macrophages were then co-cultured with isolated oxidized human RBCs for up to 24 hours. mRNA levels were measured by qPCR for *Nrf2*, *HO-1/HMOX1*, and Ferritin heavy chain. mRNA levels were quantified by quantitative real-time PCR in triplicates, and the results are expressed as the mean of mRNA levels normalized to β2-microglobulin ± SD. **P<0.01, ***P<0.001 vs. 0h.
**B) Heme concentrations before and after adding 0.5% Oxidized RBCs (oxiRBCs) in THP-1-RBC Co-culture experiments.** This figure shows the heme concentrations measured before and after a 24-hour co-culture of THP-1 macrophages with 0.5% oxiRBCs. Heme concentrations did not change significantly during the experiment. Heme was quantified as described in the Methods section using triplicates, and results are represented as mean values. No significant differences were observed before and after erythrophagocytosis.
**C) *HO-1/HMOX1* mRNA expression in THP-1 macrophages treated with non-oxidized RBCs, oxidized RBCs, CuSO₄, and Glucose Oxidase (GOX).** This figure depicts *HO-1/HMOX1* mRNA expression in THP-1 macrophages following treatment with non-oxidized RBCs for 24h, oxidized RBCs, for 24h the oxidation agent CuSO₄, for 2h and various concentrations of glucose oxidase (GOX) for 24h (+: 1:2.7x10^6^, ++: 1:9x10^5^, +++: 1:3x10^5^, ++++: 1:1x10^5^). Notably, CuSO₄, under the conditions used to oxidize RBCs, did not induce *HO-1/HMOX1* expression, whereas GOX, a specific H₂O₂ source, significantly upregulated *HO-1/HMOX1* mRNA.


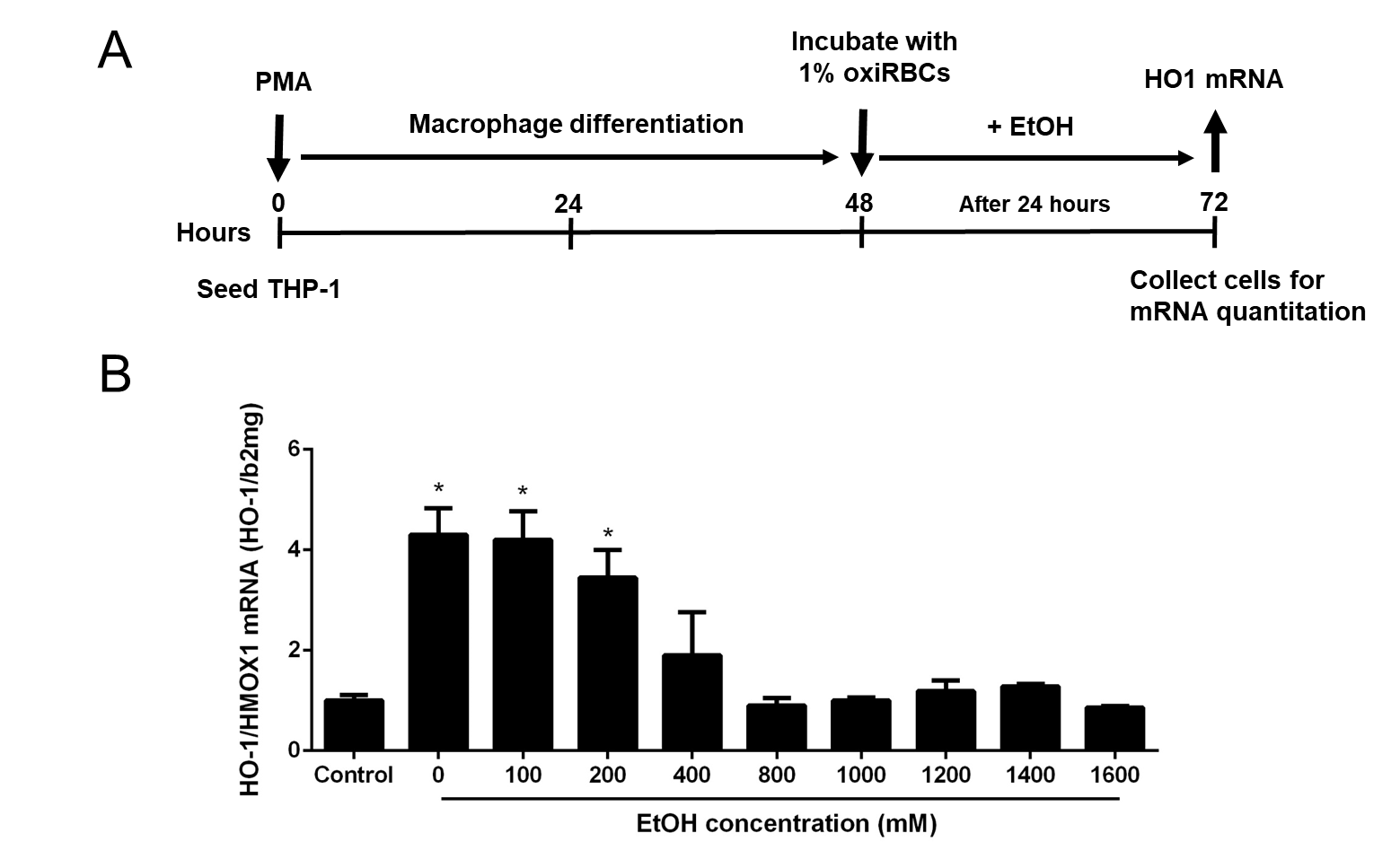

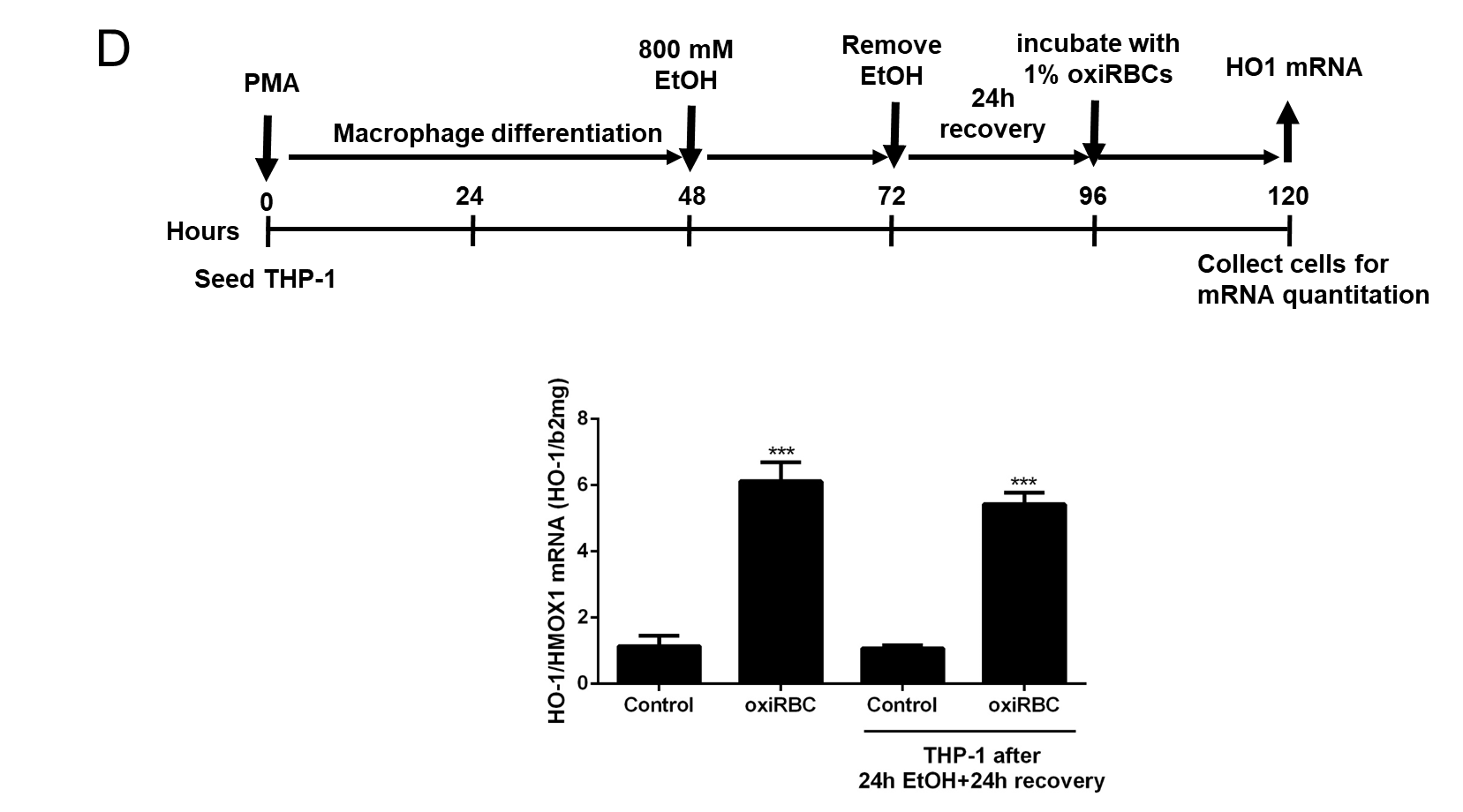

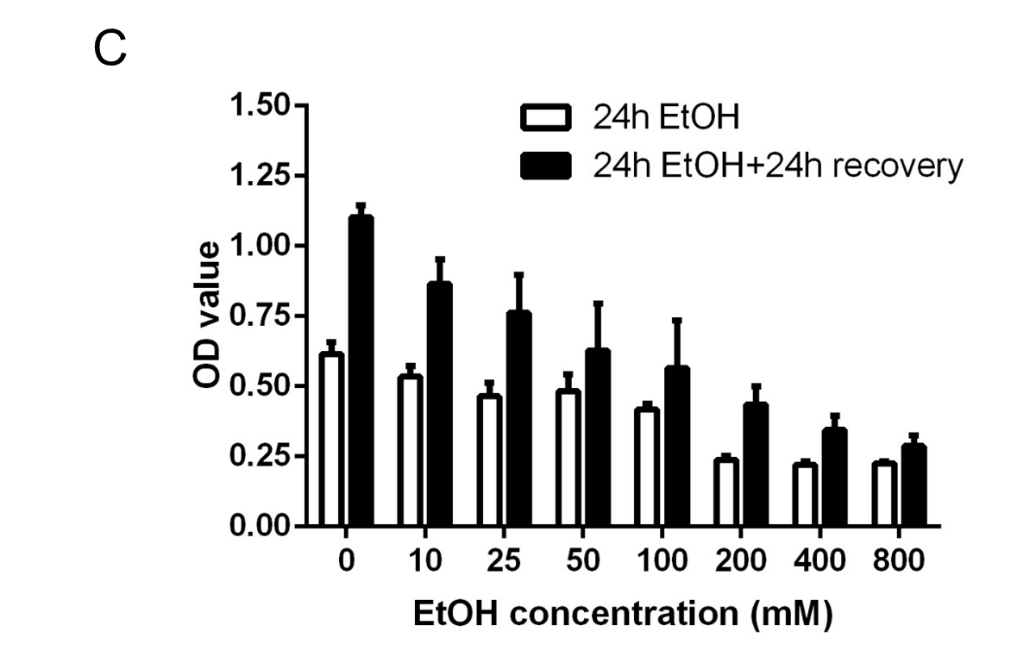


**Suppl. Fig. 7: Ethanol is able to block erythrophagocytosis through general toxicity.**

**A) Experimental design to assess the inhibition of erythrophagocytosis by ethanol.** THP-1 macrophages were differentiated over 48 hours and subsequently exposed to oxidized RBCs pretreated with copper sulfate for 120 minutes. The co-cultured RBC-THP-1 macrophages were then treated with increasing concentrations of ethanol for 24 hours. RBCs treated with CuSO₄ for 2 hours served as a positive control. **B) HO-1/HMOX1 mRNA expression** levels were increasingly inhibited starting at 200 mM ethanol. The inhibition was reversible, as THP-1 macrophages regained their ability to phagocytose after a 24-hour recovery period (refer to Fig. 6). **C) Toxic effects of ethanol on macrophages were assessed using the MTT assay.** THP-1 macrophages were seeded into 96-well plates and exposed to various concentrations of ethanol for 24 hours. A second group was allowed to recover for 24 hours post-alcohol treatment. The number of viable cells was then quantified by MTT assay, with data represented as the mean ± SD from triplicates. In the ethanol-treated group without recovery, cell viability was significantly reduced at 200 mM ethanol. Recovery for 24 hours allowed THP-1 macrophages to recover and grow even at high ethanol concentrations. Results are shown as mean ± SD. **D)** **THP-1 macrophages after 24h regeneration.** THP-1 macrophages were seeded into 12-well plates, treated with 800 mM ethanol for 24 hours, and then allowed to recover for 24 hours. Subsequently, they were co-cultured with oxidized RBCs for an additional 24 hours, and *HO-1/HMOX1* mRNA expression was measured. Oxidized RBCs induced erythrophagocytosis in THP-1 macrophages even after ethanol treatment with a recovery period. First control and oxiRBC columns on the left side are without EtOH treatment and recovery. For **B** and **D**, *HO-1/HMOX1* mRNA levels were quantified using real-time PCR. Results are represented as mean mRNA levels normalized to β2-microglobulin ± SD. Statistical significance is indicated as *P < 0.05 and **P < 0.01 compared to the control.


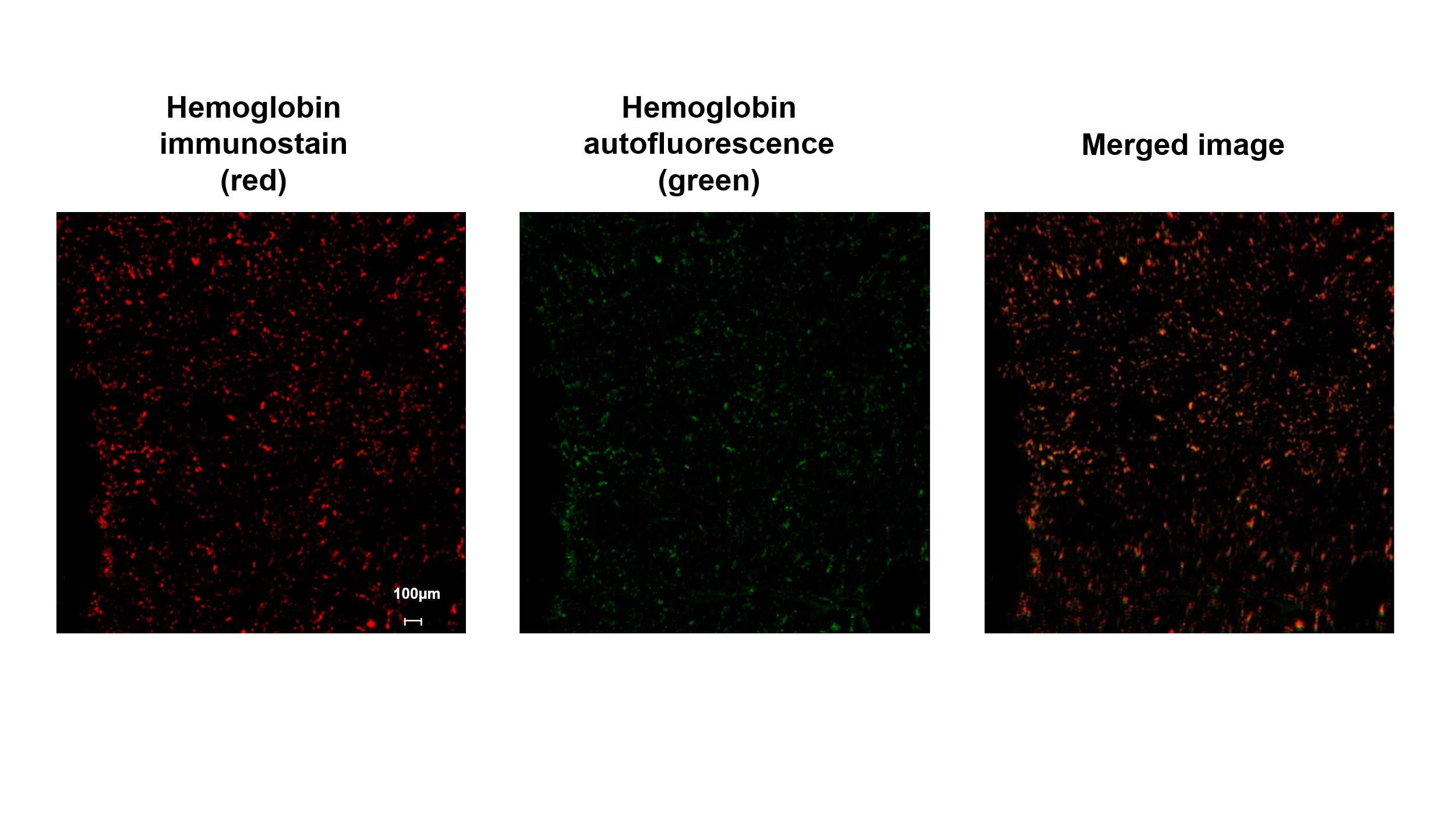


**Suppl. Fig. 8: Hemoglobin-derived autofluorescence corresponds to hemoglobin immunostaining in liver tissue.** Mice were treated with the hemolytic agent phenylhydrazine for 48 hours. Liver sections were stained with a recombinant rabbit monoclonal antibody against hemoglobin alpha (Invitrogen, SN70-09) and visualized using Alexa Fluor 647-conjugated donkey anti-rabbit IgG (Jackson ImmunoResearch, 711-605-152). The left panel shows the immunostaining signal (red), the middle panel displays hemoglobin autofluorescence detected in the Alexa Fluor 488 channel (green), and the right panel presents the merged image, demonstrating strong colocalization and confirming that autofluorescence reliably marks hemoglobin deposits in liver tissue. Imaging was performed using a ZEISS Axio Scan.Z1 microscope (Carl Zeiss Microscopy GmbH, Germany) with a 20× objective. No autofluorescence signal was observed in liver sections from untreated control mice (see Fig. 3E, second panel from top left).
